# Serotype distribution of invasive pneumococcal disease from countries of the WHO Africa, Americas, Eastern Mediterranean, South-East Asia, and Western Pacific regions: a systematic literature review from 2010 to 2021

**DOI:** 10.3389/fpubh.2024.1402795

**Published:** 2024-07-10

**Authors:** Mark A. Fletcher, Derek Daigle, Mariana Siapka, Marc Baay, Germaine Hanquet, Graciela del Carmen Morales

**Affiliations:** ^1^Pfizer Vaccines Emerging Markets, Medical Affairs, Paris, France; ^2^Pfizer Vaccines Emerging Markets, Medical Affairs, New York, NY, United States; ^3^P95 Epidemiology & Pharmacovigilance, Leuven, Belgium

**Keywords:** pneumococcal conjugate vaccines, IPD (invasive pneumococcal disease), serotype, undifferentiated, surveillance, World Health Organization, age groups, immunization programs

## Abstract

**Background:**

Most publications on invasive pneumococcal disease (IPD) serotype distribution are from about 20 countries (Australia, Canada, China, European Union members, Japan, New Zealand, South Korea, and USA). Here, we reviewed the literature among underrepresented countries in the Americas (AMRO), Africa (AFRO), Eastern Mediterranean (EMRO), South-East Asia (SEARO), and Western Pacific (WPRO) WHO regions.

**Methods:**

We performed a systematic review of the most recent IPD serotype surveillance publications (from 01/01/2010 to 31/12/2021, Medline/Embase) in those WHO regions. Selection criteria were delineated by contemporality, within-country geographical scope, and number of samples. Reported serotype distributions for each country were stratified by age group, pneumococcal conjugate vaccine (PCV) serotype category (considering undifferentiated serotypes), and PCV program period (pre-PCV, intermediate, or PCVhv [higher valency PCV formulation]). Pre-PCV period pooled data estimated PCV serotype category distribution by age group across WHO regions, while for the PCVhv period, country-level dataset tables were prepared.

**Results:**

Of 2,793 publications screened, 107 were included (58 pediatric, 11 adult, 37 all ages, and one comprising every age group). One-third of eligible countries (51/135) published serotype distribution, ranging from 30 to 43% by WHO region. Considering number of samples per WHO region, a few countries prevailed: AMRO (Brazil), AFRO (South Africa, Malawi, and Burkina Faso), and WPRO (Taiwan). In the pre-PCV period, PCV13 formulation serotypes predominated: ranging from 74 to 85% in children and 58–86% in adults in the different WHO regions. The PCVhv period represented half of the most recent IPD surveillance by countries (26/51). Undifferentiated serotypes represented >20% of IPD from most countries (34/51).

**Conclusion:**

Ubiquity of undifferentiated serotypes among the publications could constrain estimates of PCV program impact and of serotype coverage for newer PCVhv formulations; consequently, we recommend that countries favor techniques that identify serotypes specifically and, rather than reporting PCV formulation serotype distributions, provide serotype results individually.

**Systematic review registration:**

The protocol has been prospectively registered at PROSPERO, identifier: CRD42021278501. https://www.crd.york.ac.uk/prospero/display_record.php?RecordID=278501.

## Introduction

*Streptococcus pneumoniae* infection can lead to invasive pneumococcal disease (IPD) where the pneumococcus invades otherwise sterile body sites such as the bloodstream or the central nervous system, associated with clinical presentations like sepsis, pneumonia, or meningitis. Childhood immunization against *S. pneumoniae* is recommended by the World Health Organization (WHO) (1). Pneumococcal conjugate vaccines (PCVs) are licensed and available in childhood national immunization programs (NIPs) of up to 160 countries. These pediatric PCV NIPs have helped to prevent pneumococcal disease and save lives around the world (2, 3).

Timely surveillance of predominant IPD serotypes is essential for evaluation of the status of a PCV NIP, where changes in vaccine-serotype (VT) IPD serotype distribution, in the background of lower IPD incidence after PCV introduction, can help to substantiate serotype-specific disease burden declines through both direct effects on vaccinated children and indirect effects on unvaccinated children and adults (4, 5). The proportion of non-vaccine serotype (NVT) nasopharyngeal carriage and transmission might increase (6, 7), which can be attributed to serotype replacement due to the uncovering of minor serotypes obscured by the previously dominant vaccine serotypes in the nasopharynx or by the phenomenon of “capsular switch” (i.e., a change in the expressed capsular serotype), and NVT nasopharyngeal carriage consequently could lead to an increase in NVT IPD. Extensive IPD serotype distribution information is available from countries mostly located in North America, the European Union, and in Asia (namely, Japan, South Korea, and China, as well as New Zealand and Australia). Elsewhere, recently published publications with serotype distribution surveillance are limited, although low- and middle-income countries represent a substantial proportion of the IPD burden worldwide (8). For instance, between 2012 and 2017, four largely populated countries — Democratic Republic of the Congo, India, Nigeria, and Pakistan — accounted for half of the global pneumococcal disease deaths (9). Absent or incomplete serotype distributions may hamper informed evaluations of pneumococcal disease burden.

The objective of this systematic review was to describe, among countries that tend to be underrepresented in the international IPD surveillance literature, the most up to date serotype distributions based on criteria of contemporality, geographic scope, and sample size.

## Methods

We conducted a systematic literature review of observational studies as well as the control arm of interventional studies that reported an IPD serotype distribution from 135 countries of the WHO regions of Africa, Americas (except Canada and USA), Eastern Mediterranean, South-East Asia, and the Western Pacific Regions (except Australia, China, Japan, New Zealand, and South Korea). The protocol has been prospectively registered at PROSPERO [CRD42021278501]. We searched MEDLINE via PubMed, EMBASE, LILACS, and CABI Direct (Global Health) for relevant articles published between 1 January 2010 and 31 December 2021. Search terms were as follows: (((((*streptococcus pneumoniae*) OR (pneumococ*)) AND (serotyp*)) AND ((((((((invasive pneumococcal disease) OR (“IPD”)) OR ((((bacteremia) OR (bacteremic) OR (invasive)) AND (pneumonia)) OR (pleural effusion)) OR (empyema)) OR (peritonitis)) OR (osteoarticular infection)) OR (hemolytic uremic syndrome)) OR (arthritis)) OR (endocarditis))) AND ((“2010/01/01”[Date – Publication]: “3,000”[Date – Publication]))) NOT ((immunogenicity) OR (immune response)). The systematic literature review was performed using the PRISMA (Preferred Reporting Items for Systematic Reviews and Meta-Analyses) guidelines (10). Reference lists from eligible articles were also searched to identify other relevant studies.

### Inclusion criteria

Publications describing pneumococcal studies had to meet the following inclusion criteria by (a) population and (b) outcome: (a) persons of any age and gender participating in studies performed in one of the selected countries; and (b) serotype distribution of laboratory-confirmed IPD, as established by microbiological culture, molecular detection assay, and/or antigen-based testing.

All *S. pneumoniae* serotypes were captured as reported by the investigators. Publications in the following languages were included: English, French, Spanish, Portuguese, or Dutch.

### Exclusion criteria

Publications from countries in the WHO European Region (EURO), Canada, the United States of America, Japan, South Korea, China (including Hong Kong), New Zealand, or Australia were excluded. Publications that only included complicated pneumococcal pneumonia (e.g., empyema) were also excluded.

### Study selection process and data extraction

Two researchers screened publications obtained through the electronic searches based on title and abstract, using DistillerSR [Version 2.35. Evidence Partners. https://www.evidencepartners.com], and they conducted a full-text review to make a final selection of eligible publications for data extraction.

Selection of the most recent publications per country was based on an algorithm using three criteria: contemporality, scope, and size. This selection process was performed separately for the three age groups (pediatric, adult, and all ages).

- Contemporality: all publications with a study period that began in 2010 or later, or that included the year 2015, were included. If for a given country and age group, no publication was available in this period, then the most recently available publication was included according to the selection criteria of “Scope” and “Size.”
- Scope: national (country-wide) studies were prioritized over regional (i.e., region of a country), which were prioritized over sub-regional.
- Size: total number of cases: “100+” prioritized over “51–99,” which was prioritized over “25–50.”

Note that any single publication that included at least 25 cases in total with a study period that began in 2010 or later, or that included the year 2015, would be included.

A single reviewer extracted the data. Quality control of data extraction was performed by a second reviewer through re-extraction of 10% of the publications. Uncertainties were settled through discussion with the entire study team. The reasons for exclusion were documented in accordance with PRISMA guidelines (10) and included: (a) inappropriate outcome, (b) inappropriate period, (c) inappropriate design, (d) small study size, (e) data not available, and (f) duplicate publication.

Study variables for data extraction were country (i.e., location where the study was performed), study period, study design, age range of the population, geographical setting (e.g., national, regional, or subregional), clinical presentation, sample source, number of samples, PCV vaccination program during the study period, laboratory methods for detection and serotyping (classical Quellung serotyping or molecular methods that include PCR and whole genome sequencing), and serotype distribution (reported as serotype proportions or serotype-specific incidence rates).

### Quality assessment

Quality assessment of the selected publications was done using an adaption of the Newcastle-Ottawa Scale (NOS) for assessing the quality of cohort and case–control studies (11) and the National Institute of Health (NIH) checklist for before-after and cross-sectional studies (12). The quality of the extracted publications was scored as “good”, “fair”, or “poor”.

### Analysis

As detailed in Table 1, each reported serotype was categorized in one of three manners: as “serotype clearly identifiable” that could be unambiguously included in a pneumococcal conjugate vaccine serotype formulation category (i.e., PCV13, PCV20non13, or NonPCV20); as “undifferentiated”; or as “serotyping not done”.

**Table 1:** Definitions for pneumococcal conjugate vaccine (PCV) formulation serotype coverage estimates.

PCV formulation serotypes	Definition
Serotype clearly identifiable	
PCV13 serotypes	Serotypes 1, 3, 4, 5, 6A, 6B, 7F, 9V, 14, 18C, 19A, 19F, and 23F
PCV20nonPCV13 serotypes	Serotypes 8, 10A, 11A, 12F, 15B/C, 22F, and 33F
NonPCV20 serotypes	Any other individual serotype identified
Undifferentiated	Serotype results that do not allow one to distinguish between PCV20 (PCV13 or PCV20nonPCV13) and nonPCV20 serotypes (e.g., cases reported as 6A/B/C/D or “other serotypes”). This includes results where serotypes are described as “undetermined” (i.e., in which serotyping was attempted but results are described as non-typeable, untypeable, or negative), “not confirmed,” “not specified,” or “non-classified.” This should be distinguished from a “serotyping not done” category. (See below.)
Serotyping not done	“Serotyping not done” includes descriptions such as “not tested,” “untyped,” “insufficient sample,” or “unidentified,” as well as when reported as “unknown (i.e., such as reporting serotype data for only a fraction of isolates).

15B/C: serotype 15C was included as PCV20nonPCV13 as 15B and 15C were often reported together as 15B/C in many studies.

Serotype 15C was included in the PCV20non13 serotype formulation category because serotypes 15B and 15C were often described together as 15B/15C. The serotype distribution was reported as proportions (%) calculated by the number of samples with “serotype clearly identifiable” (i.e., a specific serotype or serogroup) divided by the total number of samples that had been serotyped; consequently, the denominator included “undifferentiated” samples. In contrast, samples categorized as “serotyping not done” were not included in the denominator.

We classified serotype distribution by age range into three age groups. The “pediatric” age group included cases whose age was in the range between 0 to <18 years. The “adult” age group included cases whose age was ≥18 years of age. The third group, labeled “all ages”, included publication reporting that did not differentiate between pediatric and adult cases.

We classified PCV program periods as “pre-PCV”, “intermediate”, or “higher valency PCV” (PCVhv, PCV10 or PCV13). In a pre-PCV period, serotype data was collected before an NIP started (even if PCV was available in the private market), whereas for a PCVhv period a single higher valency PCV was used in the NIP. Nonetheless, certain PCV programs could not be unambiguously identified as pre-PCV or PCVhv, and these periods were classified as “intermediate”. For instance, an intermediate period could correspond to a PCV7 period, before PCVhv was introduced. Publications that reported across years representing a mix of PCV program periods that could not be distinguished, but included PCVhv, were classified as “multiple”.

The analysis was descriptive. We determined from each publication the serotype classification (e.g., serotype clearly identifiable, undifferentiated, or serotyping not done), and we then calculated serotype proportions by country (classified by WHO region), by years of surveillance, by age group, and by PCV program period. IPD serotype results were stratified by PCV program during the study surveillance period, regardless of any subsequent changes to the PCV program after the closing year of the study. From the pre-PCV period, we pooled IPD serotype proportions across the same country (classified by WHO region), age group, and PCV program period. In the pre-PCV period, we identified through pooling the top five ranking nonPCV20 serotypes (by WHO region and age group), and this analysis was performed in R version 4.1.0 to avoid skewing by smaller-sized studies. In the intermediate and PCVhv periods, there was heterogeneity across the countries—by the vaccine used (PCV10 or PCV13), by the transition history of any preceding PCVs introduced in the national program, by the PCV uptake, and by the maturity of the program since introduction. Consequently, for intermediate or PCVhv period results, we presented the distribution of individual serotypes in tabular format by country, publication by publication.

## Results

### Study selection

**Figure 1:**
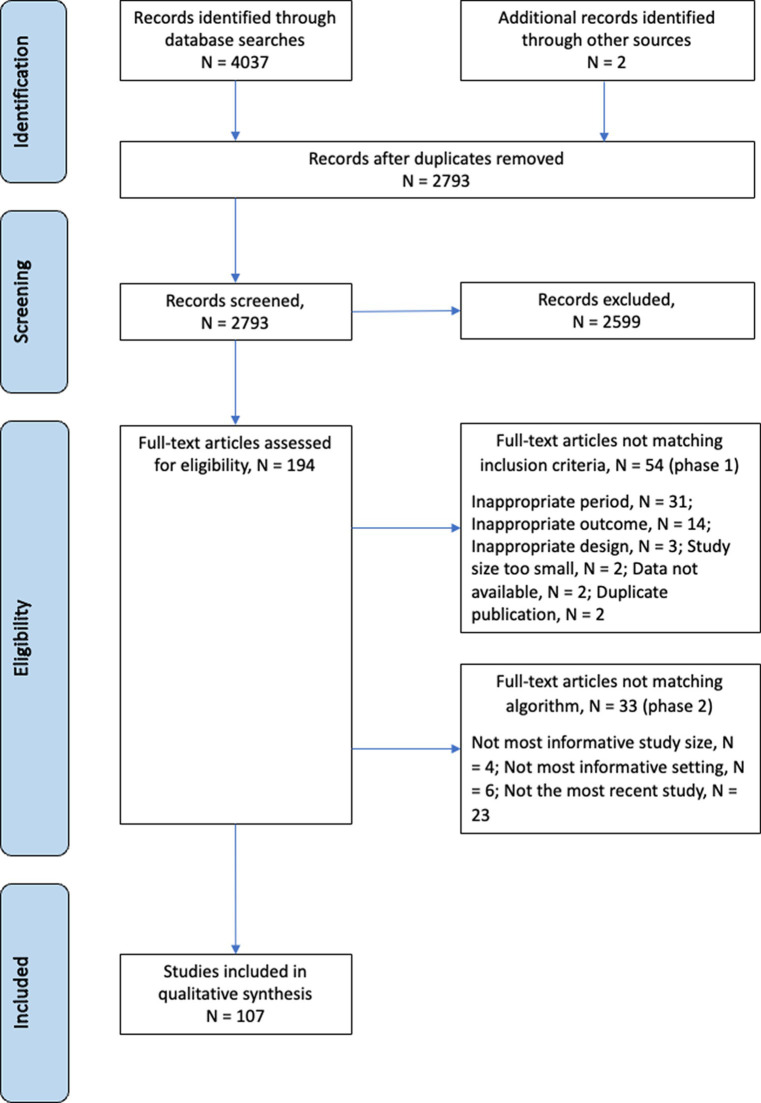
PRISMA diagram.

We identified 2,793 publications during the selection process (Figure 1). Of the 2,793 publications, 194 were assessed for eligibility by full-text review: 54 met the exclusion criteria and 33 did not adhere to the contemporality, scope, or size criteria of the selection algorithm. In total, 107 publications were retained. Only four of 107 publications reported incidences at the serotype level (13–16).

See Supplementary Table S1 for the 107 included publications and Supplementary Table S2 for the 87 publications excluded after full text review. The quality assessment scored 43 publications as “good”, 51 as “fair”, and 13 as “poor” (Supplementary Table S1, final column). In general, for publications that scored good or fair, the risk of bias was deemed acceptable. Although studies with a poor score were perceived to have a high risk of bias, they were kept when they provided the only available data for a given country.

Full tables of individual serotypes are available in the Supplement (Supplementary Tables S3–S5).

### Distribution of publications

**Figure 2:**
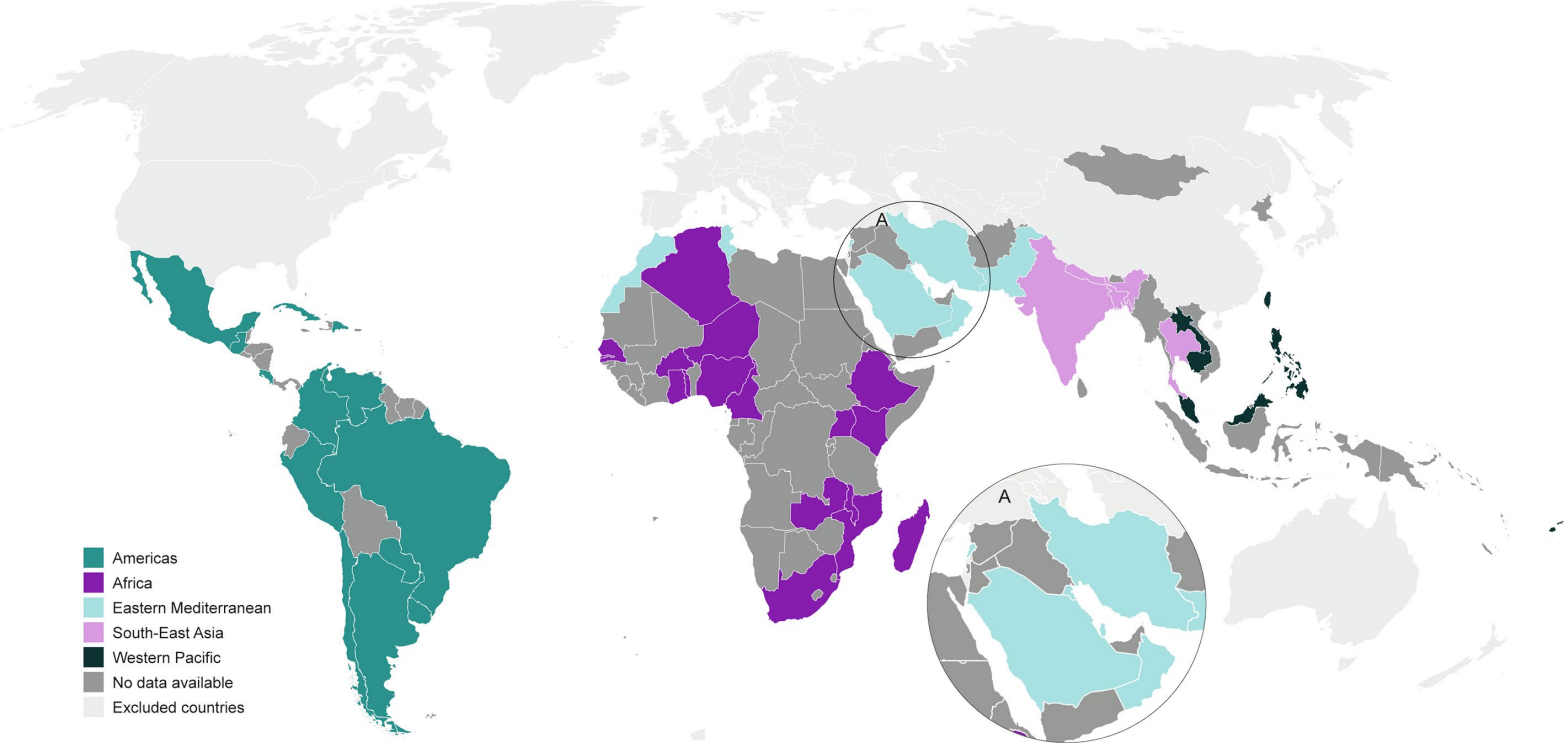
Global map of countries providing the most recent IPD serotype surveillance publications (from 2010 to 2021) in these WHO regions.

These publications were distributed by WHO region (Figure 2) as follows: 26 publications from AMRO (13, 17–42), 32 publications from AFRO (14, 15, 43–71), 17 publications from EMRO (16, 72–87), 13 publications from SEARO (88–100), and 19 publications from WPRO (100–119).

Among the 135 eligible countries across the five WHO regions, we identified publications from 51 countries. By proportion of countries per region, the publications represented 30 to 43% of countries for each WHO region: 39% (13 of 33) from AMRO (excluding Canada and USA); 38% (18 of 47) from AFRO; 43% (9 of 21) from EMRO; 36% (4 of 11) from SEARO; and 30% (7 of 23) from WPRO (excluding Japan, South Korea, China/Hong Kong, New Zealand, and Australia).

When looking at the age groups, 58 publications reported on pediatric, 11 on adult, and 37 on all-ages populations, while one publication reported separately on each of the three age groups (81). Further stratification within the pediatric group showed that one publication reported on 0–2-year-olds (28), 11 publications reported on 0–5-year-olds, including the publication reporting on 0–2-year-olds, while the remaining 47 publications reported on 0–18-year-olds.

In terms of the PCV program period, 47 publications reported from the pre-PCV period, five publications from the intermediate period and 40 publications from the PCVhv period. Fifteen publications that reported across a mix of PCV program periods that could not be distinguished but included PCVhv were classified as “multiple”. The publication breakdown by WHO region is shown in Table 2.

**Table 2:** Distribution of most recent publications over PCV program periods, by WHO region.

Region	Pre-PCV	Intermediate	PCVhv	Multiple
AMRO	7	2	16^a^	1
AFRO	9	0	16^b^	7
EMRO	9	1	4	3
SEARO	13	0	0	0
WPRO	9	2	4	4

AMRO – the Americas, AFRO – Africa, EMRO – Eastern Mediterranean, SEARO – South-East Asia, WPRO – Western Pacific, PCV – pneumococcal conjugate vaccine, Pre-PCV – the period before a NIP started (even if PCV was available in the private market), Intermediate – a period that could not be identified as pre-PCV or PCVhv, PCVhv – a period in which a single higher valency PCV was used in the NIP, Multiple – a time period covering at least two of the PCV program periods, for which the data could not be separated.

a One study reporting from nine different countries.

b One study reporting from three different countries.

In the 26 PCVhv period country programs, 11 countries were based on PCV10 and 15 countries on PCV13. The use of PCV10 was reported in AMRO (Brazil, Chile, Colombia, and Paraguay), AFRO (Ethiopia, Kenya, Madagascar, Mozambique and Zambia), and EMRO (Morocco and Pakistan), but not in WPRO. PCV13 was used in AMRO (Argentina, Dominican Republic, Mexico, and Uruguay), AFRO (Burkina Faso, Cameroon, Gambia, Ghana, Niger, South Africa, and Togo), EMRO (Kuwait, and Oman), and WPRO (Singapore, and Taiwan).

### Serotype distributions and pre-PCV period

Study duration in the pre-PCV period ranged widely, between studies that began surveillance in 1995 (Gambia, Malawi), 1996 (Guatemala), or 1997 (Singapore) and other studies covering a more recent period, such as 2016–2019 (India) or 2017–2019 (Iran).

**Figure 3:**
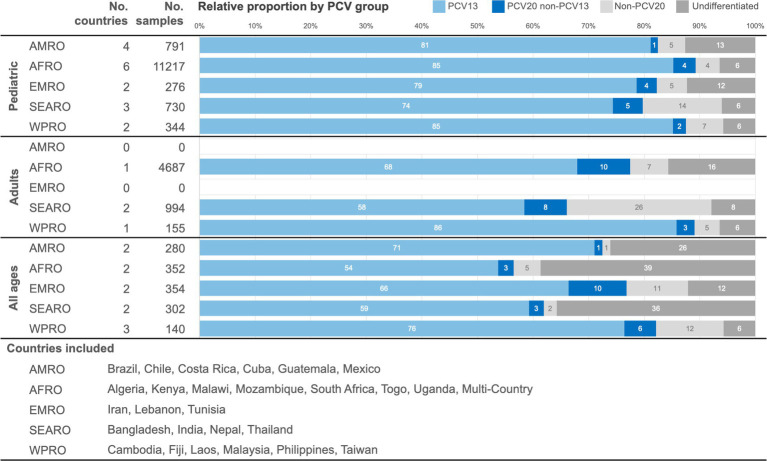
Pooled distribution by PCV formulation serotype coverage categories in the pre-PCV period by age, presented by WHO region. AMRO – the Americas, AFRO – Africa, EMRO – Eastern Mediterranean, SEARO – South-East Asia, WPRO – Western Pacific, PCV – pneumococcal conjugate vaccine.

In the pre-PCV period, the pooled distribution of serotype categories is presented by WHO region (Figure 3). The proportion of PCV13 serotypes was consistently large across regions, ranging from a potential serotype coverage of 74 to 85% in the pediatric age group, from 58 to 86% in adults, and from 54 to 76% in the all-ages population. The proportion of PCV20non13 serotypes ranged from 1 to 5% in the pediatric age group, from 3 to 10% in adults, and from 1 to 10% in the all-ages population. Finally, the proportion of nonPCV20 serotypes ranged from 4 to 14% in the pediatric age group, from 5 to 26% in adults, and from 1 to 12% in the all-ages population. A substantial proportion of serotype distribution data fell in the “undifferentiated” category, ranging from 1.2% (India) to 55.4% (Togo).

The top five nonPCV20 serotypes from the pre-PCV period pooled data differed by WHO region and by age group (Table 3). Overall, the most frequently identified nonPCV20 serotypes in the pre-PCV period were 6C, 9N, 15A, 23A, and 13. In detail, the results for the nonPCV20 serotypes by pooled proportion of all serotypes or by pooled proportion of nonPCV20 serotypes for all regions combined were as follows: 6C (1.3% of all, 16.9% of nonPCV20), 9N (1.2% of all, 16.0% of nonPCV20), 15A (1.0% of all, 12.6% of nonPCV20), 23A (1.0% of all, 12.3% of nonPCV20), and 13 (0.6% of all, 7.9% of nonPCV20).

**Table 3:** Top 5 non-PCV20 serotypes, in descending prevalence, in the pre-PCV period.

Pre-PCV period	Pediatric age group					Adult age group					All ages				
	1st	2nd	3rd	4th	5th	1st	2nd	3rd	4th	5th	1st	2nd	3rd	4th	5th
AMRO	2	6C	7C	35B	15A	9N	6C	15A	16F	18B	9N	6C	15A	16F	38
AFRO	35B	32A	9A	6C	15A	16	9N	25	13	n.a.	38	20	9N	17F	34
EMRO	15A	34	35B	6C	31	n.a.	n.a.	n.a.	n.a.	n.a.	23B	9N	16F	23A	35B
SEARO	12A	24F	20	9N	10F	25A	23A	13	38	31	33B	17F	16F	24B	35B
WPRO	18F	2	10C	11B	11F	23A	33C	6F	n.a.	n.a.	24F	34	38	23A	23B

n.a., not available.

### Serotype distributions, intermediate, and PCVhv periods

For the intermediate and PCVhv periods, the serotype distributions are presented by publication in a country-level dataset table in Table 4. A substantial proportion of the serotype distribution from the intermediate and PCVhv period in AFRO and EMRO fell in the “undifferentiated” category. Higher numbers of publications and countries in the intermediate or PCVhv period with a proportion > 20% of “undifferentiated” serotypes were found in AFRO, [58.3% (14 of 24) of publications and 80.0% (12 of 15) of countries] and in EMRO, (50.0% (5 of 10) of publications and 33.3% (2 of 6) of countries), compared to the other WHO regions [14.3% (5 of 35) of publications and 30.8% (4 of 13) of countries].

**Table 4:** Distribution by PCV formulation serotype coverage categories in the intermediate and PCVhv periods.

Region	Country	Age group	PCV period	PCV in program (year of introduction)	Study ID	Study period	PCV13	PCV20nonPCV13	nonPCV20	Undifferentiated	Total
							%	%	%	%	*N*
AMRO	Argentina	Pediatric	PCV-hv	PCV13 (2012)	3860	2012 – 2013	62.7	11.9	7.5	13.4	67
					4035	2012 – 2013	79.4	–	–	20.6	563
					1270	2015 – 2017	34.3	21.0	32.7	12.0	434
		Adults	PCV-hv	PCV13 (2012)	1245	2013 – 2017	43.1	27.6	4.6	24.8	791
	Brazil	Pediatric	PCV-hv	PCV10 (2010)	1828	2010 – 2012	72.0	9.8	17.8	0.3	325
					1450	2011 – 2015	51.6	–	–	48.4	62
					1270	2015 – 2017	46.7	18.1	29.7	5.4	441
		Adults	PCV-hv	PCV10 (2010)	197	2013 – 2015	51.0	23.5	17.6	7.8	102
		All ages	PCV-hv	PCV10 (2010)	748	2010 – 2012	61.6	14.5	19.5	4.4	159
					121	2014 – 2015	47.5	19.9	25.0	7.4	1630
					120	2017 – 2019	44.6	17.2	36.8	1.5	2402
	Chile	Pediatric	PCV-hv	PCV10 (2011)	1127	2012 – 2012	60.4	–	1.1	38.5	91
					1270	2015 – 2017	52.7	14.6	28.2	4.5	404
	Colombia	Pediatric	PCV-hv	PCV10 (2011)	1270	2015 – 2017	66.8	4.8	26.1	3.1	352
		Adults	PCV-hv	PCV10 (2011)	4034	2012 – 2019	48.1	14.2	37.7	–	310
		All ages	PCV-hv	PCV10 (2011)	3267	2012 – 2017	56.9	11.4	31.6	0.1	842
	Dominican Republic	Pediatric	PCV-hv	PCV13 (2013)	1270	2015 – 2017	67.2	3.4	19.0	10.3	58
		All ages	PCV-hv	PCV13 (2013)	3480	2013 – 2016	89.7	–	–	10.3	39
	Mexico	Pediatric	PCV-hv	PCV13 (2011)	1270	2015 – 2017	41.2	18.3	32.0	8.5	153
	Paraguay	Pediatric	PCV-hv	PCV10 (2011)	1270	2015 – 2017	67.9	10.1	13.8	8.3	109
	Peru	Pediatric	Intermediate	PCV7 (2009)	646	2009 – 2011	84.5	3.4	10.3	–	58
		All ages	Intermediate	PCV7 (2009)	2136	2010 – 2011	64.6	15.2	15.2	2.5	79
	Uruguay	Pediatric	PCV-hv	PCV13 (2010)	1270	2015 – 2017	43.7	19.5	29.9	6.9	87
		All ages	PCV-hv	PCV13 (2010)	348	2011 – 2012	46.1	–	–	53.9	356
	Venezuela	Pediatric	Multiple	pre-PCV, PCV13 (2014)	1270	2006 – 2017	90.6	–	–	9.4	309
AFRO	Burkina Faso	Pediatric	PCV-hv	PCV13 (2013)	3356	2014^a^	88.0	–	–	12.0	25
						2017^b^	50.0	–	7.1	42.9	14
		All ages	PCV-hv	PCV13 (2013)	2320	2014 – 2015	72.7	8.1	3.3	15.8	877
					3355	2016 – 2017	56.0	0.5	2.0	41.4	739
	Cameroon	Pediatric	Multiple	pre-PCV, PCV13 (2011)	1493	2010 – 2016	55.2	27.6	17.2	–	29
			PCV-hv	PCV13 (2011)	2536	2015 – 2018	35.8	17.0	30.2	17.0	53
	Ethiopia	All ages	PCV-hv	PCV10 (2011)	1001	2018 – 2019	37.1	5.7	28.6	28.6	35
	Gambia	Pediatric	Multiple	Pre-PCV, PCV7 (2009), PCV13 (2011)	2575	1995 – 2016	63.1	17.7	19.2	–	203
	Ghana	All ages	PCV-hv	PCV13 (2012)	2445	2015 – 2016	69.5	10.2	3.4	16.9	59
					1497	2015 – 2017	71.5	–	–	28.5	137
	Kenya	Pediatric	PCV-hv	PCV10 (2011)	400	2012 – 2016	26.8	–	–	73.2	82
	Madagascar	Pediatric	PCV-hv	PCV10 (2012)	4041	2013 – 2018	19.5	1.1	37.9	41.4	87
	Malawi	Pediatric	Multiple	Pre-PCV, PCV13 (2011)	2575	1995 – 2016	73.0	6.6	20.4	–	226
		All ages	Multiple	Pre-PCV, PCV13 (2011)	1407	2006 – 2018	54.5	–	–	45.5	1594
	Mozambique	Pediatric	PCV-hv	PCV10 (2013)	785	2013 – 2014	62.0	2.0	–	36.0	50
					2870	2013 – 2015	71.0	–	–	29.0	69
					695	2014 – 2015	70.0	3.3	20.0	6.7	30
	Niger	All ages	PCV-hv	PCV13 (2014)	2958	2016 – 2018	42.4	5.3	14.1	38.2	170
	Nigeria	Pediatric	Multiple	Pre-PCV, PCV10 (2014)	1073	2010 – 2016	50.0	–	3.6	46.4	28
	Senegal	Pediatric	Multiple	Pre-PCV, PCV13 (2013)	3365	2010 – 2016	91.4	–	–	6.9	58
	South Africa	Pediatric	PCV-hv	PCV13 (2011)	2575	2013 – 2014	28.2	31.6	39.9	0.3	291
		All ages	PCV-hv	PCV13 (2011)	3614	2012	53.6	20.4	12.3	13.9	1631
	Togo	Pediatric	PCV-hv	PCV13 (2014)	1111	2014 – 2016	60.0	–	–	40.0	5
	Zambia	Pediatric	PCV-hv	PCV10 (2013)	4033	2014 – 2019	52.0	2.0	–	46.0	50
	Multi-country	Pediatric	Multiple	Pre-PCV, PCV-hv	3261	2010 – 2016	33.5	4.1	–	62.4	370
EMRO	Kuwait	All ages	PCV-hv	PCV13 (2010)	2771	2010 – 2013	28.9	26.7	33.3	11.1	45
	Morocco	Pediatric	PCV-hv	PCV13 (2011), PCV10 (2012)	1807	2011 – 2014	95.9	–	–	4.1	68
	Oman	Pediatric	PCV-hv	PCV13 (2012)	1290	2014 – 2016	25.7	–	22.9	51.4	35
		Adults	PCV-hv	PCV13 (2012)	1290	2014 – 2016	40.0	–	8.3	51.7	60
		All ages	PCV-hv	PCV13 (2012)	1290	2014 – 2016	32.4	–	8.1	59.5	37
	Pakistan	Pediatric	PCV-hv	PCV10 (2012)	916	2013 – 2017	21.7	–	–	78.3	92
		All ages	Multiple	Pre-PCV, PCV10 (2012)	3275	2005 – 2013	42.3	5.4	14.4	37.8	111
	Qatar	All ages	Intermediate	PCV7 (2005)	302	2005 – 2009	77.9	7.4	12.3	2.5	122
	Saudi Arabia	Pediatric	Multiple	PCV7 (2009), PCV13 (2010)	19	2009 – 2012	84.6	2.6	6.4	6.4	78
		All ages	Multiple	Pre-PCV, PCV7 (2009), PCV13 (2010)	2772	2000 – 2016	91.7	7.9	9.0	8.7	277
WPRO	Cambodia	Pediatric	Multiple	Pre-PCV, PCV13 (2015)	3514	2012 – 2018	86.4	–	13.6	–	22
	Singapore	Adults	PCV-hv	PCV13 (2011)	554	2012 – 2017	71.4	11.1	12.7	4.8	63
		All ages	Multiple	Pre-PCV, PCV13 (2011)	689	1997 – 2013	70.5	7.4	16.9	5.2	757
	Taiwan	Pediatric	Intermediate	PCV7 (2005)/PCV13 (2015)^c^	191	2013 – 2014	80.5	5.5	13.9	–	36
			PCV-hv	PCV13 (2015)	2590	2015 – 2017	39.0	9.3	51.7	–	205
		Adults	Multiple	PCV7 (2005)/PCV13 (2015)^c^	599	2011 – 2015	66.0	11.3	18.0	4.7	150
			PCV-hv	PCV13 (2015)	189	2017 – 2020	44.7	9.7	41.8	3.8	237
		All ages	Intermediate	PCV7 (2005)/PCV13 (2015)^c^	3399	2012 – 2014	76.9	7.7	15.4	1.0	104
			Multiple	PCV7 (2005)/PCV13 (2015)^c^, PCV13 (2015)	182	2013 – 2017	57.8	17.5	23.8	1.0	206
			PCV-hv	PCV13 (2015)	3702	2016 – 2018	44.4	16.2	33.3	6.1	99

PCV13: PCV7: 4, 6B, 9V, 14, 19F, 18C, 23 and PCV13-additional (1, 5, 7F, 3, 6A, 6A/6B, 19A). PCV20: PCV20-additional (22F, 33F, 8, 10A, 11A, 12F, 15B, 15C, 15B/C, 15B/15C). nonPCV20: PPSV23-unique (2, 9N, 17F, 20, 20A, 20B) and NVST. Undifferentiated: Not differentiated and undetermined. PCV-hv – higher valent PCV, i.e., PCV10 or PCV13 as last PCV in NIP. Due to data extraction from figures with percentages, the sum of percentages may not always match 100%. See Supplementary Table S1 for the citation of each Study ID publication.

a Hounde & Kaya regions.

b Titao region.

c Catch-up for older children (2–5 in 2013, 1–2 in 2014).

For the PCVhv period, most publications reported between 40 to 300 isolates by study, although reported sample size varied from less than 10 to greater than 6,000. From the pediatric age group, EMRO surveillance is limited to 205 pediatric samples in our selection, while SEARO did not report any results. Adult surveillance is even more scarce, limited to 6 publications, based on only 63 samples from SEARO, 60 samples from EMRO, and no data from AFRO.

A disbalance per region existed in the number of samples available, and a few countries predominated in each region. AMRO contributed more than 10,000 samples, and Brazil contributed 5,100 samples or half of the AMRO samples, with the remaining half distributed between nine other countries. AFRO contributed nearly 7,000 samples. South Africa (1922 samples), Malawi (1820 samples), and Burkina Faso (1,655 samples), each contributed individually about one-quarter of AFRO samples, with the remaining quarter (1,515 samples) distributed between 12 other countries. WPRO contributed nearly 2000 samples, while the main contributor was Taiwan (1,037 samples, or 55% of the total). EMRO contributed 925 samples (evenly distributed across six countries).

The range of proportions of PCV13 serotypes in the PCVhv period varied slightly by age group: pediatric, between 20% (Madagascar) and 96% (Morocco); adults, from 40% (Oman) to 71% (Singapore); and in all ages group, from 29% (Kuwait) to 90% (Dominican Republic). The PCV20non13 serotype proportion values differed by country, ranging from 1% (Madagascar) to 32% (South Africa) in pediatric, 10% (Taiwan) to 28% (Argentina) in adults, and 1% (Burkina Faso) to 27% (Kuwait) in all ages. Across the countries, particular nonPCV20 serotypes predominated according to the age group: pediatric (23A, 6C, 15A, 23B, and 16F), adults (23A, 15A, 9N, 6C, and 29), and in all ages (6C, 9N, 15A, 23A and 20). The range of nonPCV20 serotypes differed by country, from 1% (Chile) to 52% (Taiwan) in the pediatric age group, from 5% (Argentina) to 42% (Taiwan) in adults, and from 2% (Burkina Faso) to 37% (Brazil) in the all-ages group.

## Discussion

The global map shows that there is still an IPD serotype distribution world to conquer (Figure 2). Our overview of the published studies available, within the WHO regions of Africa, Americas, Eastern Mediterranean, South-East Asia, and Western Pacific, revealed that published IPD serotype distributions were lacking from two-thirds of the 135 eligible countries; depending on the region, 57 to 70% of countries lacked published IPD serotype distributions in the literature. Furthermore, half of the published studies provided IPD serotyping results that would not be considered as current, given that they represented only the pre-PCV or intermediate periods. Even in WHO regions that appear to have IPD serotype results available, such as AMRO, AFRO, and WPRO, often the region is overrepresented by a few countries, while many countries in the same WHO region are not represented in the IPD surveillance literature. This does not seem to be unique to the WHO regions under review, as even for Europe national data is unavailable or incomplete (8).

The 74 to 85% proportion of pediatric PCV13 serotypes in the pre-PCV period pooled data estimations is in line with findings from other systematic reviews and multicenter studies that described in the pre-PCV10 or pre-PCV13 periods the pediatric serotype distributions in Latin America [73 to 88% (20)], Africa [81% (120)], or South-East Asia [70 to 80% (121)], while the proportion reported from these studies was even higher in East Asia and South-East Asia [92 to 93% in Taiwan and Singapore (122)]. The high proportion of IPD caused by PCV13 serotypes in the pre-PCV period would support the 2019 WHO recommendation to include PCVs in childhood NIPs worldwide (1). All of the four countries most affected by pneumococcal deaths, for instance, introduced a higher valent PCV into state or national immunization programs (Pakistan, PCV10 in 2012; the Democratic Republic of the Congo, PCV13 in 2013; Nigeria, PCV10 in 2014; and India, PCV13 in 2017). While serotype distribution results were available for Nigeria [pediatric (63)] and Pakistan [pediatric (82) and all ages (83)], there were no publications available on PCVhv period serotype distribution in the Democratic Republic of the Congo or India.

The PCVhv period represents half of the recently available study data (26 of 51 countries, 40 of 107 publications). From the pediatric groups, in particular, PCVhv period surveillance was available from all regions except SEARO that only published IPD serotype distribution results for the pre-PCV period. The proportion of PCV13 serotypes in the PCVhv period varied widely by country, in contrast to the consistently large proportions of PCV13 serotypes in the pediatric age group during the pre-PCV period data estimations pooled by region. We believe that the difference between countries is explained by the length of surveillance of these PCVhv programs. Most of our included publications only report from the initial years of an NIP, that is, the first 1 to 6 years after a PCV10 or PCV13 program implementation. Nonetheless, in publications from our review from the same country that reported successive PCV13 periods in the same population, the proportion of pediatric PCV13 serotypes gradually declined over time, for instance, falling from 88 to 56% in Burkina Faso (2011–2017) (45), from 54 to 36% in Cameroon (2011–2018) (48), and from 90 to 25% in Taiwan (2010–2017) (117) (Table 4). Furthermore, with the surveillance periods of more than 8 years after implementation that are typical of surveillance from Europe or the USA, PCV13 serotypes ultimately accounted for 23 and 22%, respectively, of all IPD in the latest periods (123, 124).

From the country-level dataset tables within the WHO regions of Africa, Americas, Eastern Mediterranean, South-East Asia, and Western Pacific, PCV20non13 serotype proportions during the PCVhv period in the pediatric age group ranged from 1 to 32%. These values overlap with those reported from a global systematic literature review that included 26 studies from the Americas and 64 from Europe, also looking at pediatric age groups, where PCV20non13 serotypes accounted for 28% of IPD (125).

Beyond the PCV20 serotypes, nonPCV20 serotypes in the PCVhv period ranged from 1 to 52% in the pediatric age group, from 5 to 42% in the adult age group, and from 1 to 27% in the all-ages group. Like the pre-PCV period pooled data estimations, the most prevalent serotypes varied by region and by age group, although serotypes 6C, 15A, and 23A were among the most frequent in varying order across regions or age groups.

Strikingly, the proportion of undifferentiated serotype cases from the PCVhv period ranges from 0 to 78%, depending on the WHO region. Moreover, the studies where undifferentiated serotypes represented greater than 20% of the reported distribution were substantial in AFRO (58% of publications, 80% of countries) and in EMRO (50% of publications, 33% of countries). There could be two explanations for these high proportions. First, investigators occasionally reported the IPD serotyping outcomes only by PCV formulation, for instance as PCV13 or nonPCV13, in which case nonPCV13 would qualify as “undifferentiated” because individual PCV20non13 or nonPCV20 serotypes are not “clearly identifiable”. Note that this reporting style is not limited to publications from AFRO and EMRO (6, 126). Second, there may be economic limitations: the cost of Quellung reagents for testing may be prohibitive, while the application of PCR primers may not precisely identify a serotype.

By contrast to the pediatric age group, PCVhv period country-level results for adults are not available in certain regions, such as in AFRO, while the only a few adult IPD serotype results were available from EMRO (<100) or WPRO (<450). Although the evidence required for policymaking on childhood immunization programs explains the pediatric focus, our findings suggest a lack of surveillance systems or serotyping capacity dedicated to adult IPD in AFRO, EMRO, and WPRO.

A major strength of our review is that it identified 51 countries from five WHO regions to provide an analysis of the most recently available serotype distributions by PCV program period and by age group. We followed a systematic process (based on pre-established criteria of contemporality, geographic scope, and sample size) that allowed us to include the most recent publications from all countries, including occasionally, if no more recent publications were available, publications that were published earlier than the contemporality criteria or with sizes as low as 25 samples.

This review has limitations. First, this is not a systematic review of all publications from those regions and countries, as we focused this overview on the most recently available publications of serotype distribution in countries from these WHO regions. Second, two-thirds of the 135 countries that were reviewed across the five WHO regions lacked IPD serotype publications. Third, a few countries in each region had the largest data sets, dominating the published literature in the PCVhv period, such as Brazil for AMRO, Burkina Faso, Malawi, and South Africa for AFRO, and Taiwan for WPRO. Consequently, PCVhv period results were either lacking from many countries or among other countries in the same region based on fewer samples. Fourth, about half of the countries with published results provided surveillance from a PCVhv period; consequently, the most recently available published information from the other countries represented in the surveillance literature was restricted to the pre-PCV or intermediate period. A fifth limitation is the lack of incidence outcomes that prevents us from determining the absolute disease burden. A final limitation is the high proportion of undifferentiated serotype reporting, as explained above, that is due to surveillance settings where the applied serotyping method did not allow the IPD serotype to be clearly identifiable.

## Conclusion

The WHO recommends that, rather than awaiting local surveillance results, countries with no serotype distribution surveillance in place take into consideration in the decision to introduce a PCV program the information from neighboring countries that have similar disease burden and socioeconomic or demographic patterns, which could include “sustained, high-quality sentinel and population-based surveillance for pneumococcal disease” and periodic cross-sectional surveys of nasopharyngeal carriage to provide “an indication of potential indirect effects of vaccination” (1). Nonetheless, once an NIP is in place, individual countries may expect national surveillance results to take evidence-based decisions about PCV program impact and about the possible transition to higher valency PCVs. Although about half of the recently published IPD surveillance in the five WHO regions covered by this review is from a higher valency PCV program period, the ubiquity of undifferentiated serotype reporting could hinder accurate estimates of serotype coverage for future higher valency formulations. Our review points to the need to enhance serotype reporting worldwide, first, by providing results by each individual serotype rather than based only on a calculated PCV formulation serotype coverage estimate and, second, by favoring serotyping methods that avoid “undifferentiated” serotype outcomes.

## Data availability statement

The original contributions presented in the study are included in the article/Supplementary material, further inquiries can be directed to the corresponding author.

## Author contributions

MAF: Conceptualization, Data curation, Funding acquisition, Methodology, Supervision, Validation, Writing – original draft. DD: Conceptualization, Data curation, Supervision, Validation, Writing – review & editing. MS: Data curation, Formal analysis, Investigation, Methodology, Validation, Visualization, Writing – review & editing. MB: Data curation, Formal analysis, Investigation, Methodology, Validation, Visualization, Writing – original draft. GH: Data curation, Formal analysis, Investigation, Methodology, Supervision, Validation, Visualization, Writing – original draft. GC: Conceptualization, Data curation, Supervision, Validation, Writing – review & editing.
