# Supplementary material for: Serotype distribution of invasive pneumococcal disease from countries of the WHO Africa, Americas, Eastern Mediterranean, South-East Asia, and Western Pacific regions: a systematic literature review from 2010 to 2021
Source: Front Public Health. 2024 Jul 10;12:1402795. doi: 10.3389/fpubh.2024.1402795 (PMC11266301; doi:10.3389/fpubh.2024.1402795)
Supplement: Supplementary file 1 [file Data_Sheet_1.docx]

Supplemental files to:

Serotype distribution of invasive pneumococcal disease from countries of the WHO Africa, Americas, Eastern Mediterranean, South-East Asia, and Western Pacific regions: a systematic literature review from 2010 to 2021

Mark A. Fletcher, Derek Daigle, Mariana Siapka, Marc Baay, Germaine Hanquet, Graciela del Carmen Morales

Table of Contents

[Supplement 1. Included papers 2](#_Toc144279737)

[Supplement 2. Excluded papers 7](#_Toc144279738)

[Supplement 3. Individual serotypes by PCV period and by WHO region – pediatric 17](#_Toc144279739)

[Supplement 4. Individual serotypes by PCV period and by WHO region – adults 21](#_Toc144279740)

[Supplement 5. Individual serotypes by PCV period and by WHO region – all ages 23](#_Toc144279741)

[Supplement 6. Countries contributing data to figure 3. 26](#_Toc144279742)

[Supplement 7. Top 5 non-PCV20 serotypes (manuscript Table 3). 27](#_Toc144279743)

# Supplement 1. Included papers

| 19 | Al-Sheikh, Ann Lab Med, 2014 | EM | Saudi Arabia | Sub-regional | 2009 | 2012 | 78 | pediatric | poor |
| --- | --- | --- | --- | --- | --- | --- | --- | --- | --- |
| 51 | Arguedas, Vaccine, 2012 | America | Costa Rica | Regional | 2007 | 2009 | 22 | pediatric | fair |
| 52 | Arjun, Infez Med, 2020 | SEA | India | Sub-regional | 2016 | 2019 | 39 | all | poor |
| 54 | Arredondo-Garcia, J Infect Dev Ctries 2011 | America | Mexico | Sub-regional | 2002 | 2005 | 150 | pediatric | poor |
| 55 | Arushoty, Int J Infect Dis, 2019 | WP | Malaysia | National | 2014 | 2017 | 245 | pediatric | good |
| 75 | Baqui, PLoS One, 2020 | SEA | Bangladesh | Sub-regional | 2014 | 2015 | 46 | pediatric | good |
| 120 | Brandileone, Vaccine, 2021 | America | Brazil | National | 2007 | 2019 | 11380 | all | good |
| 121 | Brandileone, Vaccine, 2018 | America | Brazil | National | 2005 | 2015 | 8971 | all | good |
| 129 | Brueggemann, PLoS One, 2013 | Africa | Kenya | Sub-regional | 1994 | 2008 | 1100 | pediatric | fair |
| 155 | Capeding, Pediatr Infect Dis J, 2013 | WP | Philippines | Regional | 2007 | 2009 | 47 | pediatric | fair |
| 179 | Chen, CH, Vaccine, 2019 | WP | Taiwan | Regional | 2012 | 2016 | 58 | pediatric | good |
| 182 | Chen, YY, Emerg Infect Dis, 2020 | WP | Taiwan | Regional | 2013 | 2017 | 206 | all | good |
| 183 | Chhetri, Kathmandu Univ Med J (KUMJ), 2011 | SEA | Nepal | Sub-regional | 2007 | 2009 | 42 | pediatric | poor |
| 189 | Chien, J Glob Antimicrob Resist, 2021 | WP | Taiwan | National | 2017 | 2020 | 237 | all | good |
| 191 | Cho, Pediatr Infect Dis J, 2017 | WP | Taiwan | Regional | 2010 | 2015 | 498 | pediatric | good |
| 197 | Christophe, J Med Microbiol, 2018 | America | Brazil | Sub-regional | 2013 | 2015 | 102 | adult | good |
| 210 | Cohen, PLoS One, 2015 | Africa | South Africa | National | 2003 | 2008 | 3953 | adult | fair |
| 221 | Cornick, Emerg Infect Dis, 2011 | Africa | Malawi | Regional | 2004 | 2006 | 176 | pediatric | good |
| 286 | Dullius, J Bras Pneumol, 2018 | America | Brazil | Sub-regional | 2005 | 2016 | 118 | adult | poor |
| 300 | Elmdaghri, Vaccine, 2012 | EM | Morocco | Sub-regional | 1994 | 2010 | 187 | pediatric | fair |
| 302 | ElShafie, Infect Drug Resist, 2016 | EM | Qatar | Sub-regional | 2005 | 2009 | 134 | all | fair |
| 343 | Gaensbauer, Pediatr Infect Dis J, 2016 | America | Guatemala | Sub-regional | 1996 | 2007 | 452 | pediatric | fair |
| 348 | Garcia, PLoS One, 2014 | America | Uruguay | National | 2003 | 2012 | 1887 | pediatric | good |
| 377 | Gopi, Indian J Med Microbiol, 2016 | SEA | India | Sub-regional | 2010 | 2011 | 40 | pediatric | fair |
| 394 | Habibi, Infect Drug Resist, 2020 | EM | Iran | Sub-regional | 2017 | 2019 | 80 | all | fair |
| 400 | Hammitt, Lancet, 2019 | Africa | Kenya | Sub-regional | 1999 | 2016 | 667 | all | good |
| 409 | Hanna-Wakim, Vaccine, 2012 | EM | Lebanon | National | 2005 | 2011 | 257 | all | fair |
| 451 | Houri, Int J Infect Dis, 2017 | EM | Iran | Sub-regional | 2013 | 2016 | 585 | pediatric | fair |
| 499 | Jain, Indian J Med Microbiol, 2020 | SEA | India | Sub-regional | 2012 | 2016 | 25 | all | fair |
| 554 | Kim, Vaccine, 2020 | Multi - SEA | Thailand (multi-country) | Regional | 2012 | 2017 | 33 | adult | poor |
| 554 | Kim, Vaccine, 2020 | Multi - WP | Singapore (multi-country) | Regional | 2012 | 2017 | 63 | adult | poor |
| 596 | Lee HY, J Microbiol Immunol Infect, 2018 | WP | Taiwan | Regional | 2011 | 2013 | 268 | all | good |
| 598 | Lee MC, J Microbiol Immunol Infect, 2019 | WP | Taiwan | Regional | 2010 | 2016 | 46 | pediatric | fair |
| 599 | Lee MC, J Microbiol Immunol Infect, 2020 | WP | Taiwan | Sub-regional | 2011 | 2015 | 150 | adult | fair |
| 646 | Luna-Muschi, Epidemiol Infect, 2019 | America | Peru | Sub-regional | 2006 | 2011 | 159 | pediatric | good |
| 664 | Manoharan, Lancet Infect Dis, 2017 | SEA | India | National | 2011 | 2015 | 226 | pediatric | fair |
| 689 | Martinez-Vega, PLoS One, 2019 | WP | Singapore | Sub-regional | 1997 | 2013 | 889 | all | good |
| 695 | Massora, Vaccine, 2019 | Africa | Mozambique | Regional | 2012 | 2015 | 40 | pediatric | good |
| 728 | Moghnieh, Vaccine, 2019 | EM | Lebanon | Sub-regional | 2006 | 2015 | 103 | all | fair |
| 739 | Moore, Am J Trop Med Hyg, 2010 | WP | Laos | Sub-regional | 2003 | 2009 | 33 | all | fair |
| 748 | Mott, Int J Infect Dis, 2014 | America | Brazil | Sub-regional | 2010 | 2012 | 159 | all | fair |
| 751 | Mousavi, Iran J Microbiol, 2013 | EM | Iran | Sub-regional | 2011 | 2012 | 36 | pediatric | fair |
| 785 | Nhantumbo, BMC Microbiol, 2016 | Africa | Mozambique | Regional | 2013 | 2014 | 50 | pediatric | fair |
| 836 | Parra, PLoS One, 2014 | America | Colombia | National | 2005 | 2010 | 1775 | all, per age | good |
| 860 | Phongsamart, Hum Vaccin Immunother, 2014 | SEA | Thailand | Regional | 2009 | 2012 | 238 | all | fair |
| 916 | Riaz, Int J Infect Dis, 2019 | EM | Pakistan | Regional | 2013 | 2017 | 92 | pediatric | fair |
| 1001 | Sharew, Infect Drug Resist, 2021 | Africa | Ethiopia | Regional | 2018 | 2019 | 57 | all | fair |
| 1015 | Sigaúque, PLoS One, 2018 | Africa | Mozambique | Sub-regional | 2001 | 2012 | 543 | pediatric | good |
| 1042 | Soto-Nogueron, Int J Infect Dis, 2016 | America | Mexico | National | 2000 | 2014 | 126 | pediatric | good |
| 1046 | Srifeungfung, Hum Vaccin Immunother, 2014 | SEA | Thailand | Regional | 2005 | 2012 | 157 | adult | fair |
| 1073 | Tagbo, Clin Infect Dis, 2019 | Africa | Nigeria | Regional | 2010 | 2016 | 71 | pediatric | fair |
| 1111 | Tsolenyanu, Clin Infect Dis, 2019 | Africa | Togo | Sub-regional | 2010 | 2016 | 66 | pediatric | fair |
| 1127 | Valenzuela, Rev Chilena Infectol, 2014 | America | Chile | National | 2007 | 2012 | 91 | pediatric | good |
| 1216 | Yasin, Vaccine, 2011 | WP | Malaysia | National | 2008 | 2009 | 216 | all | fair |
| 1244 | Ziane, Front Microbiol, 2016 | Africa | Algeria | Regional | 2010 | 2014 | 80 | pediatric | fair |
| 1245 | Zintgraff, Rev Argent Microbiol, 2020 | America | Argentina | National | 2013 | 2017 | 791 | adult | good |
| 1257 | Abdoli, Ir J Med Sci, 2020 | EM | Iran | Regional | 2014 | 2018 | 106 | pediatric | good |
| 1270 | Agudelo, Lancet Infect Dis, 2021 | America | Argentina (multi-country) | National | 2006 | 2017 | 434 | pediatric | good |
| 1270 | Agudelo, Lancet Infect Dis, 2021 | America | Brazil (multi-country) | National | 2006 | 2017 | 441 | pediatric | good |
| 1270 | Agudelo, Lancet Infect Dis, 2021 | America | Chile (multi-country) | National | 2006 | 2017 | 404 | pediatric | good |
| 1270 | Agudelo, Lancet Infect Dis, 2021 | America | Colombia (multi-country) | National | 2006 | 2017 | 352 | pediatric | good |
| 1270 | Agudelo, Lancet Infect Dis, 2021 | America | Cuba (multi-country) | National | 2006 | 2017 | 320 | pediatric | good |
| 1270 | Agudelo, Lancet Infect Dis, 2021 | America | Dominican Rep (multi-country) | National | 2006 | 2017 | 58 | pediatric | good |
| 1270 | Agudelo, Lancet Infect Dis, 2021 | America | Mexico (multi-country) | National | 2006 | 2017 | 153 | pediatric | good |
| 1270 | Agudelo, Lancet Infect Dis, 2021 | America | Paraguay (multi-country) | National | 2006 | 2017 | 109 | pediatric | good |
| 1270 | Agudelo, Lancet Infect Dis, 2021 | America | Uruguay (multi-country) | National | 2006 | 2017 | 87 | pediatric | good |
| 1270 | Agudelo, Lancet Infect Dis, 2021 | America | Venezuela (multi-country) | National | 2006 | 2017 | 309 | pediatric | good |
| 1290 | Al-Jardani, Int J Infect Dis, 2019 | EM | Oman | National | 2014 | 2016 | 132 | all | good |
| 1407 | Bar-Zeev, Lancet Global Health, 2021 | Africa | Malawi | National | 2006 | 2018 | 605 | all | good |
| 1450 | Berezin, Vaccine, 2020 | America | Brazil | Regional | 2005 | 2015 | 260 | pediatric | good |
| 1493 | Boula, Clinical Infectious Diseases, 2019 | Africa | Cameroon | Sub-regional | 2010 | 2016 | 31 | pediatric | poor |
| 1497 | Bozio, PLoS ONE, 2018 | Africa | Ghana | Regional | 2015 | 2017 | 137 | pediatric | fair |
| 1628 | Chi HC, J Microbiol Immunol Infect, 2018 | WP | Taiwan | Regional | 2001 | 2015 | 307 | adult | good |
| 1807 | Diawara, International Journal of Infectious Diseases, 2015 | EM | Morocco | Sub-regional | 2007 | 2014 | 136 | pediatric | fair |
| 1828 | Domingues, The Lancet Respiratory Medicine, 2014 | America | Brazil | National | 2010 | 2012 | 316 | pediatric | fair |
| 1866 | Echaniz-Aviles, Archives of Medical Research, 2015 | America | Mexico | National | 1993 | 2012 | 1346 | pediatric | fair |
| 1985 | Ganaie, Journal of Pediatric Infectious Diseases, 2016 | SEA | India | Sub-regional | 2013 | 2015 | 1504 | pediatric | fair |
| 2007 | Gessner, Vaccine, 2012 | Africa | Multi-country | Regional | 2007 | 2009 | 282 | all | fair |
| 2094 | Haddad-Boubaker African Health Sciences, 2020 | EM | Tunisia | Regional | 2014 | 2017 | 34 | pediatric | fair |
| 2136 | Hawkins, International Journal of Medical Microbiology, 2017 | America | Peru | Sub-regional | 2006 | 2011 | 212 | pediatric | good |
| 2237 | Inghammar, Am J Trop Med Hyg, 2018 | WP | Cambodia | National | 2005 | 2014 | 26 | all | poor |
| 2278 | Jayaraman R, Journal of Microbiology, Immunology and Infection, 2019 | SEA | India | Regional | 2007 | 2017 | 408 | adult | fair |
| 2279 | Jayaraman Y, PLoS ONE, 2018 | SEA | India | Regional | 2012 | 2013 | 257 | pediatric | fair |
| 2280 | Jayaraman Y, Vaccine, 2021 | SEA | India | Regional | 2012 | 2016 | 586 | pediatric | fair |
| 2320 | Kambire, Journal of Infection, 2018 | Africa | Burkina Faso | National | 2011 | 2015 | 1734 | all | good |
| 2445 | Kwambana-Adams, BMC Infectious Diseases, 2016 | Africa | Ghana | Regional | 2015 | 2016 | 59 | all | poor |
| 2536 | Libwea, PLoS ONE, 2021 | Africa | Cameroon | Regional | 2011 | 2018 | 103 | pediatric | good |
| 2575 | Lo, The Lancet Infectious Diseases, 2019 | Africa | Gambia (multi-country) | National | 1995 | 2016 | 86 | pediatric | good |
| 2575 | Lo, The Lancet Infectious Diseases, 2019 | Africa | Malawi (multi-country) | National | 1995 | 2016 | 13 | pediatric | good |
| 2575 | Lo, The Lancet Infectious Diseases, 2019 | Africa | South Africa (multi-country) | National | 1995 | 2016 | 104 | all | good |
| 2590 | Lu, Clinical Infectious Diseases, 2019 | WP | Taiwan | National | 2013 | 2017 | 217 | pediatric | fair |
| 2768 | Moisi, PLoS ONE, 2017 | Africa | Togo | Regional | 2010 | 2013 | 1654 | all | poor |
| 2771 | Mokaddas, Expert Review of Vaccines, 2016 | EM | Kuwait | National | 2003 | 2013 | 45 | all | good |
| 2772 | Mokaddas, Vaccine, 2018 | EM | Saudi Arabia | National | 2000 | 2016 | 332 | all | good |
| 2870 | Nhantumbo, PLoS ONE, 2017 | Africa | Mozambique | Regional | 2013 | 2015 | 86 | pediatric | fair |
| 2958 | Ousmane, Vaccine, 2020 | Africa | Niger | National | 2010 | 2018 | 774 | all | good |
| 3044 | Pirez, Pediatric Infectious Disease Journal, 2017 | America | Uruguay | Sub-regional | 2005 | 2014 | 21 | pediatric | poor |
| 3188 | Russell, Pediatric Infectious Disease Journal, 2010 | WP | Fiji | National | 2004 | 2007 | 83 | all | fair |
| 3261 | Senghore, Microbial Genomics, 2021 | Africa | Multi-country | Regional | 2010 | 2016 | 185 | pediatric | good |
| 3267 | Severiche-Bueno, International Journal of Infectious Diseases, 2021 | America | Colombia | Regional | 2007 | 2017 | 1670 | all | fair |
| 3275 | Shakoor, PLoS ONE, 2014 | EM | Pakistan | Regional | 2005 | 2013 | 111 | all | good |
| 3355 | Soeters, Journal of Infectious Diseases, 2019 | Africa | Burkina Faso | National | 2016 | 2017 | 1478 | all | good |
| 3356 | Soeters, Vaccine, 2020 | Africa | Burkina Faso | National | 2011 | 2017 | 66 | all | fair |
| 3365 | Sonko, Clinical Infectious Diseases, 2019 | Africa | Senegal | National | 2010 | 2016 | 76 | pediatric | fair |
| 3399 | Su, Scientific reports, 2015 | WP | Taiwan | Sub-regional | 2012 | 2014 | 104 | pediatric | good |
| 3420 | Tabatabaei, Iranian Journal of Pediatrics, 2021 | EM | Iran | Sub-regional | 2012 | 2013 | 83 | pediatric | fair |
| 3480 | Tomczyk, BMC Infectious Diseases, 2018 | America | Dominican Rep | Regional | 2013 | 2016 | 39 | pediatric | good |
| 3486 | Torano-Peraza, Vaccimonitor, 2014 | America | Cuba | Sub-regional | 2007 | 2012 | 237 | all, per age | fair |
| 3514 | Turner, Clinical Infectious Diseases, 2020 | WP | Cambodia | National | 2012 | 2018 | 81 | pediatric | fair |
| 3613 | von Gottberg, Vaccine, 2013 | Africa | South Africa | National | 2003 | 2008 | 8674 | pediatric | fair |
| 3614 | von Gottberg, New England Journal of Medicine, 2014 | Africa | South Africa | National | 2005 | 2012 | 35192 | all | fair |
| 3702 | Wu, Frontiers in Microbiology, 2020 | WP | Taiwan | Regional | 2002 | 2018 | 1845 | all | good |
| 3855 | Morera Álvarez, Medisur, 2019 | America | Cuba | Sub-regional | 2009 | 2015 | 66 | pediatric | poor |
| 3860 | Perez, Med. infant, 2014 | America | Argentina | Sub-regional | 2008 | 2015 | 171 | pediatric | fair |
| 3866 | Rioseco, Rev. méd. Chile, 2018 | America | Chile | Sub-regional | 2010 | 2014 | 70 | adult | poor |
| 3875 | Torano-Peraza, Rev. cuba. pediatr, 2017 | America | Cuba | Sub-regional | 2013 | 2015 | 141 | pediatric | fair |
| 4033 | Yamba, J Infect Dis, 2021 | Africa | Zambia | National | 2010 | 2019 | 148 | pediatric | good |
| 4034 | Narváez, BMC Infect Dis, 2021 | America | Colombia | Sub-regional | 2012 | 2019 | 310 | adult | good |
| 4035 | Gagetti, Microb Genom, 2021 | America | Argentina | National | 1998 | 2013 | 1713 | pediatric | good |
| 4040 | Kisakye, Clin Infect Dis, 2009 | Africa | Uganda | Regional | 2001 | 2006 | 331 | pediatric | poor |
| 4041 | Raboba, J Infect Dis, 2021 | Africa | Madagascar | Sub-regional | 2012 | 2018 | 125 | pediatric | good |

# Supplement 2. Excluded papers

| Refid | Reference | Reason for exclusion |
| --- | --- | --- |
| 11 | Africano HF, Serrano-Mayorga CC, Ramirez-Valbuena PC, et al. Major Adverse Cardiovascular Events During Invasive Pneumococcal Disease Are Serotype Dependent. Clin Infect Dis. Jun 1 2021;72(11):e711-e719. doi:10.1093/cid/ciaa1427 | duplicate |
| 31 | Almazrou Y, Shibl AM, Alkhlaif R, et al. Epidemiology of invasive pneumococcal disease in Saudi Arabian children younger than 5years of age. J Epidemiol Glob Health. Jun 2016;6(2):95-104. doi:10.1016/j.jegh.2015.08.002 | inappropriate period |
| 45 | Andrade AL, Oliveira R, Vieira MA, et al. Population-based surveillance for invasive pneumococcal disease and pneumonia in infants and young children in Goiânia, Brazil. Vaccine. Feb 27 2012;30(10):1901-9. doi:10.1016/j.vaccine.2011.12.012 | inappropriate period |
| 66 | Ba ID, Ba A, Faye PM, et al. Pediatric invasive pneumococcal disease in Senegal. Med Mal Infect. Nov-Dec 2015;45(11-12):463-9. doi:10.1016/j.medmal.2015.10.007 | not most informative study setting |
| 68 | Balaji V, Jayaraman R, Verghese VP, Baliga PR, Kurien T. Pneumococcal serotypes associated with invasive disease in under five children in India & implications for vaccine policy. Indian J Med Res. Sep 2015;142(3):286-92. doi:10.4103/0971-5916.166588 | inappropriate period |
| 80 | Bautista-Márquez A, Richardson V, Ortiz-Orozco O, et al. Prevalence of pneumococcal disease, serotype distribution, and antimicrobial susceptibility in Mexican children younger than 5 years of age. Arch Med Res. Feb 2013;44(2):142-50. doi:10.1016/j.arcmed.2012.12.005 | inappropriate design |
| 95 | Benavides JA, Ovalle OO, Salvador GR, Gray S, Isaacman D, Rodgers GL. Population-based surveillance for invasive pneumococcal disease and pneumonia in infants and young children in Bogotá, Colombia. Vaccine. Aug 31 2012;30(40):5886-92. doi:10.1016/j.vaccine.2012.03.054 | inappropriate period |
| 132 | Budnik I, Sandoval A, Prado A, Labbé M, Peña A, Viviani T. [Occult bacteremia in Sótero del Río Hospital: Experience after pneumococcal conjugated vaccine]. Rev Chilena Infectol. Apr 2017;34(2):133-140. Bacteriemia oculta en niños atendidos en el Complejo Asistencial Dr. Sótero del Río: Experiencia post vacuna neumocóccica conjugada. doi:10.4067/s0716-10182017000200006 | duplicate |
| 143 | Caierão J, Hawkins P, Sant'anna FH, et al. Serotypes and genotypes of invasive Streptococcus pneumoniae before and after PCV10 implementation in southern Brazil. PLoS One. 2014;9(10):e111129. doi:10.1371/journal.pone.0111129 | not most informative study setting |
| 148 | Camacho Moreno G, Imbachi LF, Leal AL, et al. Emergence of Streptococcus pneumoniae serotype 19A (Spn19A) in the pediatric population in Bogotá, Colombia as the main cause of invasive pneumococcal disease after the introduction of PCV10. Hum Vaccin Immunother. Sep 1 2020;16(9):2300-2306. doi:10.1080/21645515.2019.1710411 | No appropriate outcome |
| 169 | Chacon-Cruz E, Rivas-Landeros RM, Volker-Soberanes ML. Early trends in invasive pneumococcal disease in children following the introduction of 13-valent pneumococcal conjugate vaccine: results from eight years of active surveillance in a Mexican hospital. Ther Adv Vaccines. Nov 2014;2(6):155-8. doi:10.1177/2051013614547199 | No appropriate outcome |
| 171 | Chacon-Cruz E, Velazco-Mendez Y, Navarro-Alvarez S, Rivas-Landeros RM, Volker ML, Lopez-Espinoza G. Pneumococcal disease: emergence of serotypes 19A and 7F following conjugate pneumococcal vaccination in a Mexican hospital. J Infect Dev Ctries. Jun 15 2012;6(6):516-20. doi:10.3855/jidc.1954 | not most informative study size |
| 176 | Charfi F, Smaoui H, Kechrid A. Non-susceptibility trends and serotype coverage by conjugate pneumococcal vaccines in a Tunisian paediatric population: a 10-year study. Vaccine. Dec 31 2012;30 Suppl 6:G18-24. doi:10.1016/j.vaccine.2012.07.017 | inappropriate period |
| 184 | Chiang CS, Chen YY, Jiang SF, et al. National surveillance of invasive pneumococcal diseases in Taiwan, 2008-2012: differential temporal emergence of serotype 19A. Vaccine. Jun 5 2014;32(27):3345-9. doi:10.1016/j.vaccine.2014.04.061 | not most recent study |
| 232 | Crowther-Gibson P, Cohen C, Klugman KP, de Gouveia L, von Gottberg A. Risk factors for multidrug-resistant invasive pneumococcal disease in South Africa, a setting with high HIV prevalence, in the prevaccine era from 2003 to 2008. Antimicrob Agents Chemother. Oct 2012;56(10):5088-95. doi:10.1128/aac.06463-11 | inappropriate period |
| 282 | dos Santos SR, Passadore LF, Takagi EH, et al. Serotype distribution of Streptococcus pneumoniae isolated from patients with invasive pneumococcal disease in Brazil before and after ten-pneumococcal conjugate vaccine implementation. Vaccine. Dec 9 2013;31(51):6150-4. doi:10.1016/j.vaccine.2013.05.042 | not most informative study setting |
| 294 | El Mdaghri N, Jilali N, Belabbes H, Jouhadi Z, Lahssoune M, Zaid S. Epidemiological profile of invasive bacterial diseases in children in Casablanca, Morocco: antimicrobial susceptibilities and serotype distribution. East Mediterr Health J. Nov 2012;18(11):1097-101. doi:10.26719/2012.18.11.1097 | study size |
| 316 | Feikin DR, Jagero G, Aura B, et al. High rate of pneumococcal bacteremia in a prospective cohort of older children and adults in an area of high HIV prevalence in rural western Kenya. BMC Infect Dis. Jun 23 2010;10:186. doi:10.1186/1471-2334-10-186 | inappropriate period |
| 370 | Gómez-Barreto D, Espinosa-Monteros LE, López-Enríquez C, Jiménez-Rojas V, Rodríguez-Suárez R. Invasive pneumococcal disease in a third level pediatric hospital in Mexico City: epidemiology and mortality risk factors. Salud Publica Mex. Sep-Oct 2010;52(5):391-7. doi:10.1590/s0036-36342010000500006 | inappropriate period |
| 432 | Hidalgo M, Santos C, Duarte C, Castañeda E, Agudelo CI. [Increase in erythromycin-resistant Streptococcus pneumoniae in Colombia, 1994-2008]. Biomedica. Mar 2011;31(1):124-31. Incremento de la resistencia a eritromicina de Streptococcus pneumoniae, Colombia, 1994-2008. doi:10.1590/s0120-41572011000100015 | inappropriate period |
| 455 | Howidi M, Muhsin H, Rajah J. The burden of pneumococcal disease in children less than 5 years of age in Abu Dhabi, United Arab Emirates. Ann Saudi Med. Jul-Aug 2011;31(4):356-9. doi:10.4103/0256-4947.83214 | No appropriate outcome |
| 456 | Hsiao HJ, Wu CT, Huang JL, et al. Clinical features and outcomes of invasive pneumococcal disease in a pediatric intensive care unit. BMC Pediatr. Jul 17 2015;15:85. doi:10.1186/s12887-015-0387-7 | not most recent study |
| 506 | Jauneikaite E, Mary Carnon Jefferies J, William Vere Churton N, Tzer Pin Lin R, Lloyd Hibberd M, Charles Clarke S. Genetic diversity of Streptococcus pneumoniae causing meningitis and sepsis in Singapore during the first year of PCV7 implementation. Emerg Microbes Infect. Jun 2014;3(6):e39. doi:10.1038/emi.2014.37 | not most recent study |
| 515 | John J, Varghese R, Lionell J, Neeravi A, Veeraraghavan B. Non-vaccine Pneumococcal Serotypes Among Children with Invasive Pneumococcal Disease. Indian Pediatr. Oct 15 2018;55(10):874-876. | not most informative study setting |
| 534 | Kattan R, Abu Rayyan A, Zheiman I, et al. Serotype distribution and drug resistance in Streptococcus pneumoniae, Palestinian Territories. Emerg Infect Dis. Jan 2011;17(1):94-6. doi:10.3201/eid1701.100886 | data not available |
| 546 | Kelly DF, Thorson S, Maskey M, et al. The burden of vaccine-preventable invasive bacterial infections and pneumonia in children admitted to hospital in urban Nepal. Int J Infect Dis. Jan 2011;15(1):e17-23. doi:10.1016/j.ijid.2010.05.021 | study size |
| 572 | Kumar KLR, Ganaie F, Ashok V. Circulating Serotypes and Trends in Antibiotic Resistance of Invasive Streptococcus Pneumoniae from Children under Five in Bangalore. J Clin Diagn Res. Dec 2013;7(12):2716-20. doi:10.7860/jcdr/2013/6384.3741 | not most recent study |
| 617 | Liao WH, Lin SH, Lai CC, et al. Impact of pneumococcal vaccines on invasive pneumococcal disease in Taiwan. Eur J Clin Microbiol Infect Dis. Apr 2010;29(4):489-92. doi:10.1007/s10096-010-0873-7 | inappropriate period |
| 691 | Marzouk M, Ferjani A, Bouafia N, Harb H, Ben Salem Y, Boukadida J. Serotype distribution and antimicrobial resistance of invasive and noninvasive pneumococcal isolates in Tunisia. Microb Drug Resist. Feb 2015;21(1):85-9. doi:10.1089/mdr.2014.0084 | No appropriate outcome |
| 735 | Moodley K, Coovadia YM, Cohen C, et al. Invasive Pneumococcal Disease in Neonates Prior to Pneumococcal Conjugate Vaccine Use in South Africa: 2003-2008. Pediatr Infect Dis J. Apr 2019;38(4):424-430. doi:10.1097/inf.0000000000002096 | inappropriate period |
| 737 | Moore CE, Giess A, Soeng S, et al. Characterisation of Invasive Streptococcus pneumoniae Isolated from Cambodian Children between 2007 - 2012. PLoS One. 2016;11(7):e0159358. doi:10.1371/journal.pone.0159358 | no appropriate outcome |
| 752 | Mueller JE, Yaro S, Ouédraogo MS, et al. Pneumococci in the African meningitis belt: meningitis incidence and carriage prevalence in children and adults. PLoS One. 2012;7(12):e52464. doi:10.1371/journal.pone.0052464 | inappropriate period |
| 789 | Nisarga R, Premalatha R, Shivananda, et al. Hospital-based surveillance of invasive pneumococcal disease and pneumonia in South Bangalore, India. Indian Pediatr. Mar 8 2015;52(3):205-11. doi:10.1007/s13312-015-0607-0 | not most recent study |
| 801 | Ochoa TJ, Egoavil M, Castillo ME, et al. Invasive pneumococcal diseases among hospitalized children in Lima, Peru. Rev Panam Salud Publica. Aug 2010;28(2):121-7. doi:10.1590/s1020-49892010000800008 | inappropriate period |
| 873 | Pinto TCA, Neves FPG, Souza ARV, et al. Evolution of Penicillin Non-susceptibility Among Streptococcus pneumoniae Isolates Recovered From Asymptomatic Carriage and Invasive Disease Over 25 years in Brazil, 1990-2014. Front Microbiol. 2019;10:486. doi:10.3389/fmicb.2019.00486 | not most recent study |
| 896 | Ramdani-Bouguessa N, Ziane H, Bekhoucha S, et al. Evolution of antimicrobial resistance and serotype distribution of Streptococcus pneumoniae isolated from children with invasive and noninvasive pneumococcal diseases in Algeria from 2005 to 2012. New Microbes New Infect. Jul 2015;6:42-8. doi:10.1016/j.nmni.2015.02.008 | not most recent study |
| 915 | Rhodes J, Dejsirilert S, Maloney SA, et al. Pneumococcal Bacteremia Requiring Hospitalization in Rural Thailand: An Update on Incidence, Clinical Characteristics, Serotype Distribution, and Antimicrobial Susceptibility, 2005-2010. PLoS One. 2013;8(6):e66038. doi:10.1371/journal.pone.0066038 | not most recent study |
| 918 | Rijal B, Tandukar S, Adhikari R, et al. Antimicrobial susceptibility pattern and serotyping of Streptococcus pneumoniae isolated from Kanti Children Hospital in Nepal. Kathmandu Univ Med J (KUMJ). Apr-Jun 2010;8(30):164-8. doi:10.3126/kumj.v8i2.3551 | inappropriate period |
| 959 | Saha SK, Hossain B, Islam M, et al. Epidemiology of Invasive Pneumococcal Disease in Bangladeshi Children Before Introduction of Pneumococcal Conjugate Vaccine. Pediatr Infect Dis J. Jun 2016;35(6):655-61. doi:10.1097/inf.0000000000001037 | not most recent study |
| 1005 | Shen CF, Wang SM, Lee KH, Ho TS, Liu CC. Childhood invasive pneumococcal disease caused by non-7-valent pneumococcal vaccine (PCV7) serotypes under partial immunization in Taiwan. J Formos Med Assoc. Sep 2013;112(9):561-8. doi:10.1016/j.jfma.2013.05.015 | not most informative study setting |
| 1022 | Silberbauer EJ, Ismail N, von Gottberg A, Hoosen AA. Serotype and antimicrobial profile distribution of invasive pneumococcal isolates in the pre-vaccine introduction era in Pretoria, South Africa, 2005 through 2009. Diagn Microbiol Infect Dis. Nov 2011;71(3):309-11. doi:10.1016/j.diagmicrobio.2011.07.006 | inappropriate period |
| 1080 | Tali-Maamar H, Laliam R, Bentchouala C, et al. Serotyping and antibiotic susceptibility of Streptococcus pneumoniae strains isolated in Algeria from 2001 to 2010. Med Mal Infect. Feb 2012;42(2):59-65. doi:10.1016/j.medmal.2011.12.001 | not most recent study |
| 1093 | Thomas K, Mukkai Kesavan L, Veeraraghavan B, et al. Invasive pneumococcal disease associated with high case fatality in India. J Clin Epidemiol. Jan 2013;66(1):36-43. doi:10.1016/j.jclinepi.2012.04.006 | inappropriate period |
| 1149 | Verani JR, Domingues CM, de Moraes JC. Indirect cohort analysis of 10-valent pneumococcal conjugate vaccine effectiveness against vaccine-type and vaccine-related invasive pneumococcal disease. Vaccine. Nov 17 2015;33(46):6145-8. doi:10.1016/j.vaccine.2015.10.007 | No appropriate outcome |
| 1224 | Yoshioka CR, Martinez MB, Brandileone MC, et al. Analysis of invasive pneumonia-causing strains of Streptococcus pneumoniae: serotypes and antimicrobial susceptibility. J Pediatr (Rio J). Jan-Feb 2011;87(1):70-5. doi:10.2223/jped.2063 | inappropriate period |
| 1327 | Alvares JR, Mantese OC, de Paula A, et al. Prevalence of pneumococcal serotypes and resistance to antimicrobial agents in patients with meningitis: Ten-year analysis. Article. Brazilian Journal of Infectious Diseases. 2011;15(1):22-27. doi:10.1590/S1413-86702011000100005 | inappropriate period |
| 1386 | Azevedo J, Dos Anjos ES, Cordeiro SM, et al. Genetic profiles and antimicrobial resistance of Streptococcus pneumoniae non-PCV10 serotype isolates recovered from meningitis cases in Salvador,Brazil. Article. Journal of Medical Microbiology. 2016;65(10):1164-1170. doi:10.1099/jmm.0.000346 | not most informative study setting |
| 1413 | Barroso DE, Godoy D, Castiñeiras TMPP, Tulenko MM, Rebelo MC, Harrison LH. β-lactam resistance, serotype distribution, and genotypes of meningitis-causing streptococcus pneumoniae, Rio de Janeiro, Brazil. Article. Pediatric Infectious Disease Journal. 2012;31(1):30-36. doi:10.1097/INF.0b013e31822f8a92 | inappropriate period |
| 1585 | Chaguza C, Cornick JE, Andam CP, et al. Population genetic structure, antibiotic resistance, capsule switching and evolution of invasive pneumococci before conjugate vaccination in Malawi. Article. Vaccine. 2017;35(35):4594-4602. doi:10.1016/j.vaccine.2017.07.009 | not most recent study |
| 1628 | Chi HC, Hsieh YC, Tsai MH, Lee CH, Kuo KC, Huang CT, et al. Impact of pneumococcal conjugate vaccine in children on the serotypic epidemiology of adult invasive pneumococcal diseases in Taiwan. J Microbiol Immunol Infect. 2018;51(3):332-6. | not most recent study |
| 1689 | Collard JM, Alio Sanda AK, Jusot JF. Determination of pneumococcal serotypes in meningitis cases in Niger, 2003-2011. Article. PLoS One. 2013;8(3):e60432. doi:10.1371/journal.pone.0060432 | not most recent study |
| 1840 | dos Santos MS, Azevedo J, Menezes AP, et al. Temporal trends and clonal diversity of penicillin non-susceptible pneumococci from meningitis cases from 1996 to 2012, in Salvador, Brazil. Article. BMC Infect Dis. Jul 30 2015;15(1):302. doi:10.1186/s12879-015-1049-y | not most recent study |
| 2006 | Gervaix A, Taguebue J, Bescher BN, et al. Bacterial meningitis and pneumococcal serotype distribution in children in Cameroon. Article. Pediatric Infectious Disease Journal. 2012;31(10):1084-1087. doi:10.1097/INF.0b013e318260552d | inappropriate period |
| 2068 | Grenón SL, Grabulosa MCS, Regueira MM, Fossati MS, von Specht MH. Pneumococcal meningitis in children under 15 years of age in misiones (Argentina). Sixteen year's epidemiological surveillance. Article. Revista Argentina de Microbiologia. 2014;46(1):14-23. doi:10.1016/S0325-7541(14)70042-2 | inappropriate period |
| 2289 | Jefferies JMC, Tee WSN, Clarke SC. Molecular analysis of streptococcus pneumoniae clones causing invasive disease in children in Singapore. Article. Journal of Medical Microbiology. 2011;60(6):750-755. doi:10.1099/jmm.0.030007-0 | inappropriate period |
| 2432 | Ktari S, Jmal I, Mroua M, et al. Serotype distribution and antibiotic susceptibility of Streptococcus pneumoniae strains in the south of Tunisia: A five-year study (2012–2016) of pediatric and adult populations. Article. International Journal of Infectious Diseases. 2017;65:110-115. doi:10.1016/j.ijid.2017.10.015 | No appropriate outcome |
| 2454 | Lai CC, Lin SH, Liao CH, Sheng WH, Hsueh PR. Decline in the incidence of invasive pneumococcal disease at a medical center in Taiwan, 2000-2012. Article. BMC Infect Dis. Feb 11 2014;14(1):76. doi:10.1186/1471-2334-14-76 | not most recent study |
| 2543 | Lin SH, Liao WH, Lai CC, et al. Comparison of clinical features, antimicrobial susceptibility, serotype distribution and outcomes of patients with hospital- and community-associated invasive pneumococcal disease. Article. International Journal of Antimicrobial Agents. 2010;36(2):119-123. doi:10.1016/j.ijantimicag.2010.04.001 | inappropriate period |
| 2697 | Medeiros MIC, Almeida SCG, Guerra M, da Silva P, Carneiro AMM, de Andrade D. Distribution of Streptococcus pneumoniae serotypes in the northeast macro-region of Sao Paulo state/Brazil after the introduction of conjugate vaccine. Article. BMC Infect Dis. Aug 25 2017;17(1):590. doi:10.1186/s12879-017-2696-y | not most recent study |
| 2698 | Medeiros MIC, Negrini BVM, Silva JM, et al. Clinical and microbiological implications of invasive pneumococcal disease in hospitalized patients (1998-2013). Article. Brazilian Journal of Infectious Diseases. 2016;20(3):242-249. doi:10.1016/j.bjid.2016.01.011 | not most recent study |
| 2707 | Meiring S, Cohen C, Quan V, et al. HIV Infection and the Epidemiology of Invasive Pneumococcal Disease (IPD) in South African Adults and Older Children Prior to the Introduction of a Pneumococcal Conjugate Vaccine (PCV). Article. PLoS One. 2016;11(2):e0149104. doi:10.1371/journal.pone.0149104 | inappropriate period |
| 2720 | Menezes APDO, Campos LC, dos Santos MS, et al. Serotype distribution and antimicrobial resistance of Streptococcus pneumoniae prior to introduction of the 10-valent pneumococcal conjugate vaccine in Brazil, 2000-2007. Article. Vaccine. 2011;29(6):1139-1144. doi:10.1016/j.vaccine.2010.12.021 | inappropriate period |
| 2773 | Molander V, Elisson C, Balaji V, et al. Invasive pneumococcal infections in Vellore, India: clinical characteristics and distribution of serotypes. Article. BMC Infect Dis. Nov 9 2013;13(1):532. doi:10.1186/1471-2334-13-532 | not most recent study |
| 2819 | Mwenda JM, Soda E, Weldegebriel G, et al. Pediatric Bacterial Meningitis Surveillance in the World Health Organization African Region Using the Invasive Bacterial Vaccine-Preventable Disease Surveillance Network, 2011-2016. Article. Clinical Infectious Diseases. 2019;69:S49-S57. doi:10.1093/cid/ciz472 | No appropriate outcome |
| 2852 | Ndlangisa KM, du Plessis M, Wolter N, et al. Population snapshot of Streptococcus pneumoniae causing invasive disease in South Africa prior to introduction of pneumococcal conjugate vaccines. Article. PLoS One. 2014;9(9):e107666. doi:10.1371/journal.pone.0107666 | inappropriate period |
| 3010 | Pérez GM, Parra A, Casimir L, et al. Invasive infections caused by Streptococcus pneumoniae in a tertiary-level children's hospital before the introduction of the conjugate vaccine. Clinical characteristics and serotypes involved. Article. Archivos Argentinos de Pediatria. 2013;111(3):202-205. doi:10.5546/aap.2013.202 | not most recent study |
| 3043 | Pírez MC, Algorta G, Cedrés A, et al. Impact of universal pneumococcal vaccination on hospitalizations for pneumonia and meningitis in children in montevideo, Uruguay. Article. Pediatric Infectious Disease Journal. 2011;30(8):669-674. doi:10.1097/INF.0b013e3182152bf1 | inappropriate period |
| 3057 | Pormohammad A, Lashkarbolouki S, Azimi T, et al. Clinical characteristics and molecular epidemiology of children with meningitis in Tehran, Iran: a prospective study. Article. New Microbes and New Infections. 2019;32doi:10.1016/j.nmni.2019.100594 | No appropriate outcome |
| 3189 | Ruvinsky RO, Regueira M, Fossati MS, et al. Surveillance of invasive in Streptococcus pneumoniae in Argentina 1994-2007: Changes in serotype distribution, serotype coverage of pneumococcal conjugate vaccines and antibiotic resistance. Article. Journal of Pediatric Infectious Diseases. 2010;5(3):263-269. doi:10.3233/JPI-2010-0261 | inappropriate period |
| 3201 | Saha SK, Al Emran HM, Hossain B, et al. Streptococcus pneumoniae serotype-2 childhood meningitis in Bangladesh: a newly recognized pneumococcal infection threat. Article. PLoS One. 2012;7(3):e32134. doi:10.1371/journal.pone.0032134 | inappropriate period |
| 3216 | Sanneh B, Okoi C, Grey-Johnson M, et al. Declining Trends of Pneumococcal Meningitis in Gambian Children after the Introduction of Pneumococcal Conjugate Vaccines. Article. Clinical Infectious Diseases. 2019;69:S126-S132. doi:10.1093/cid/ciz505 | No appropriate outcome |
| 3294 | Shibl AM, Memish ZA, Al-Kattan KM. Antibiotic resistance and serotype distribution of invasive pneumococcal diseases before and after introduction of pneumococcal conjugate vaccine in the Kingdom of Saudi Arabia (KSA). Review. Vaccine. 2012;30(SUPPL. 6):G32-G36. doi:10.1016/j.vaccine.2012.07.030 | Inappropriate design |
| 3376 | Srifeungfung S, Tribuddharat C, Comerungsee S, et al. Serotype coverage of pneumococcal conjugate vaccine and drug susceptibility of Streptococcus pneumoniae isolated from invasive or non-invasive diseases in central Thailand, 2006-2009. Article. Vaccine. 2010;28(19):3440-3444. doi:10.1016/j.vaccine.2010.02.071 | inappropriate period |
| 3402 | Su WJ, Lo HY, Chang CH, et al. Effectiveness of pneumococcal conjugate vaccines of different valences against invasive pneumococcal disease among children in Taiwan: A nationwide study. Article. Pediatric Infectious Disease Journal. 2016;35(4):e124-e133. doi:10.1097/INF.0000000000001054 | not most recent study |
| 3452 | Tempia S, Wolter N, Cohen C, et al. Assessing the impact of pneumococcal conjugate vaccines on invasive pneumococcal disease using polymerase chain reaction-based surveillance: an experience from South Africa. Article. BMC Infect Dis. Oct 26 2015;15(1):450. doi:10.1186/s12879-015-1198-z | No appropriate outcome |
| 3466 | Thoon KC, Chong CY, Tee NWS. Early impact of pneumococcal conjugate vaccine on invasive pneumococcal disease in Singapore children, 2005 through 2010. Article. International Journal of Infectious Diseases. 2012;16(3):e209-e215. doi:10.1016/j.ijid.2011.11.014 | not most recent study |
| 3665 | Wei SH, Chiang CS, Chiu CH, Chou P, Lin TY. Pediatric invasive pneumococcal disease in Taiwan following a national catch-up program with the 13-valent pneumococcal conjugate vaccine. Article. Pediatric Infectious Disease Journal. 2015;34(3):e71-e77. doi:10.1097/INF.0000000000000565 | not most recent study |
| 3725 | Yamaguchi M, Win HPM, Higashi K, et al. Epidemiological analysis of pneumococcal strains isolated at yangon children’s hospital in Myanmar via whole-genome sequencing-based methods. Article. Microbial Genomics. 2021;7(2):1-14. doi:10.1099/mgen.0.000523 | no appropriate outcome |
| 3800 | Abarca V K, Vergara F R, Tassara P E, Ibáñez W I, García B C, Potin S M. Infección neumocóccica invasora y neumonía consolidante en lactantes: Un año de vigilancia en tres centros hospitalarios chilenos. Invasive pneumococcal disease and consolidated pneumonia in infants: One year of surveillance in three Chilean health care centers. Rev chil infectol. 2008/04 2008;25(2):97-103. | inappropriate period |
| 3807 | Assandri E, Amorín B, Gesuele JP, Algorta G, Pírez MC. Enfermedad neumoccócica invasora en recién nacidos, antes y después de la vacunación universal con vacuna conjugada 7 y 13 valente en Uruguay. Pneumococcal invasive disease in newborns before and after 7-valent and 13-valent universal pneumococcal vaccination in Uruguay. Rev chil infectol. 2015/04 2015;32(2):167-174. | not most informative study size |
| 3814 | Budnik I, Sandoval A, Prado A, Labbé M, Pena A, Viviani T. Bacteriemia oculta en niños atendidos en el Complejo Asistencial Dr. Sótero del Río: Experiencia post vacuna neumocóccica conjugada. Occult bacteremia in Sótero del Río Hospital: Experience after pneumococcal conjugated vaccine. Rev chil infectol. 2017/04 2017;34(2):133-140. | not most informative study size |
| 3848 | Mattei SM, Falleiros-Carvalho LH, Cavalcante NJF. Doença pneumocócica invasiva em crianças e adolescentes soropositivos para HIV. Invasive pneumococcal disease in HIV seropositive children and adolescents. J pediatr (Rio J). 2008/06 2008;84(3):276-280. | not most informative study size |
| 3850 | Medeiros MIC, Almeida SCG, Bokermann S, Watanabe E, Guerra MLLS, Andrade Dd. Antimicrobial susceptibility of Streptococcus pneumoniae isolated from patients in the northeastern macroregion of São Paulo state, Brazil, 1998-2013. Suscetibilidade antimicrobiana de Streptococcus pneumoniae isolados de pacientes na macrorregião nordeste do estado de São Paulo, Brasil, entre 1998 e 2013. J Bras Patol Med Lab (Online). 2017/06 2017;53(3):177-182. | not most recent study |
| 4006 | Suphanklang J, Santimaleeworagun W, Thunyaharn S, Traipattanakul J. Pneumococcal meningitis at a Thai Hospital over a 10-year period: clinical outcomes, serotypes, and antimicrobial susceptibility patterns. Southeast Asian Journal of Tropical Medicine and Public Health. 2017;48(6):1281-1289. | inappropriate design |
| 4036 | du Plessis M, de Gouveia L, Freitas C, et al. The Role of Molecular Testing in Pediatric Meningitis Surveillance in Southern and East African Countries, 2008–2017. J Infect Dis. 2021;224(S3):S194-203 | No appropriate outcome |
| 4037 | Almeida SCG, Lo SW, Hawkins PA, et al. Genomic surveillance of invasive Streptococcus pneumoniae isolates in the period pre-PCV10 and post-PCV10 introduction in Brazil. Microb Genom. Oct 2021;7(10)doi:10.1099/mgen.0.000635 | No appropriate outcome |
| 4038 | Mackenzie GA, Hill PC, Jeffries DJ, et al. Impact of the introduction of pneumococcal conjugate vaccination on invasive pneumococcal disease and pneumonia in The Gambia: 10 years of population-based surveillance. The Lancet Infectious Diseases. 2021;21(9):1293-1302. | data not available |

# Supplement 3. Individual serotypes by PCV period and by WHO region – pediatric

| **PCV groups** | **Individual  serotypes** | **AMRO** | | **AFRO** | | **EMRO** | | **SEARO** | **WPRO** | |
| --- | --- | --- | --- | --- | --- | --- | --- | --- | --- | --- |
|  |  | **Pre-PCV N=6931** | **Current PCV N=3716** | **Pre-**  **PCV N=12644** | **Current PCV**  **N=642** | **Pre-PCV N=282** | **Current PCV**  **N = 230** | **Pre-**  **PCV  N=739** | **Pre-**  **PCV**  **N=226** | **Current PCV**  **N=527** |
|  |  | **%** | **%** | **%** | **%** | **%** | **%** | **%** | **%** | **%** |
| **PCV13** | ***Sub-total*** | *83.4* | *59.8* | *84.1* | *43.3* | *79.1* | *51.8* | *73.2* | *80.5* | *65.9* |
| **PCV20non13** | ***Sub-total*** | *0.5* | *9.0* | *4.2* | *14.6* | *3.5* | *0.9* | *5.3* | *3.2* | *5.1* |
| **nonPCV20** | **2** | 0.2 | 0.1 | 0.1 | 6.7 | - | - | 0.7 | 1.3 | - |
|  | **5A** | 0.0 | - | - | - | - | - | - | - | - |
|  | **6C** | 0.1 | 2.6 | 0.0 | - | 0.7 | - | 0.3 | - | 0.4 |
|  | **6C/6D** | - | 0.1 | - | - | - | - | - | - | - |
|  | **6F** | 0.0 | - | - | - | - | - | - | - | - |
|  | **7A** | - | - | 0.0 | - | 0.4 | - | - | - | - |
|  | **7B** | - | - | - | - | - | - | 0.3 | - | - |
|  | **7C** | 0.0 | 0.4 | 0.1 | 1.1 | - | - | 0.7 | - | - |
|  | **7B/7C** | - | 0.0 | - | - | - | - | - | - | - |
|  | **7B/7C. 40** | - | - | - | 0.3 | - | - | - | - | - |
|  | **9A** | - | 0.1 | 0.0 | - | - | - | 0.5 | - | - |
|  | **9L** | - | - | 0.1 | - | - | - | - | - | - |
|  | **9N** | 0.0 | 0.9 | 0.8 | 0.8 | - | - | 0.1 | - | - |
|  | **9A/9V** | - | - | - | 0.9 | 1.8 | - | - | - | - |
|  | **9L/9N** | - | - | 0.0 | - | - | 0.9 | - | - | - |
|  | **10B** | - | - | 0.0 | - | - | - | - | - | - |
|  | **10C** | - | - | - | - | - | - | - | 0.4 | - |
|  | **10F** | - | 0.2 | 0.0 | 0.3 | - | - | 1.1 | - | - |
|  | **10F/10C** | - | 0.0 | - | - | - | - | - | - | - |
|  | **10F/10C. 33C** | - | - | 0.0 | - | - | - | - | - | - |
|  | **10X** | - | - | 0.0 | - | - | - | - | - | - |
|  | **11B** | - | 0.0 | - | 0.6 | - | - | - | 0.4 | - |
|  | **11C** | - | - | 0.0 | - | - | - | - | - | 0.2 |
|  | **11D** | - | - | - | - | - | - | 0.1 | - | - |
|  | **11F** | - | - | - | - | - | - | 0.1 | 0.4 | - |
|  | **12A** | - | - | - | - | - | - | 0.3 | 0.4 | - |
|  | **12B** | - | 0.0 | 0.0 | - | - | - | - | - | - |
|  | **13** | 0.0 | 0.4 | 0.6 | 1.7 | - | - | - | 0.0 | 0.2 |
|  | **15A** | 0.0 | 1.6 | 0.1 | 1.1 | 1.8 | - | 0.5 | 1.3 | 1.7 |
|  | **15F** | 0.0 | - | - | - | - | - | 0.1 | - | - |
|  | **15A/15B/15C** | 0.1 | - | - | - | - | - | - | - | - |
|  | **15A/15F** | - | 0.0 | - | - | 0.4 | - | - | - | - |
|  | **15-non B** | - | - | - | - | - | - | - | - | 15.2 |
|  | **16** | - | - | 0.7 | - | - | - | - | - | - |
|  | **16F** | 0.0 | 0.8 | 0.2 | 2.6 | - | 1.3 | 0.3 | - | - |
|  | **17F** | - | 0.1 | 0.0 | 1.2 | - | 0.9 | 0.4 | - | - |
|  | **18A** | 0.0 | 0.0 | 0.0 | - | - | - | 0.4 | - | - |
|  | **18B** | - | 0.1 | 0.0 | - | - | - | 0.1 | - | - |
|  | **18F** | - | 0.1 | 0.0 | - | - | - | 0.5 | 1.3 | - |
|  | **19B** | - | 0.1 | 0.0 | 0.2 | - | - | 0.4 | - | 0.8 |
|  | **20** | - | 0.4 | 0.0 | - | - | - | 0.5 | - | - |
|  | **20A** | - | - | 0.0 | - | - | - | - | - | - |
|  | **20B** | - | - | 0.0 | - | - | - | - | - | - |
|  | **21** | - | 0.2 | 0.0 | 1.1 | - | - | 0.4 | - | - |
|  | **22A** | - | - | 0.0 | - | - | - | 0.1 | - | - |
|  | **23A** | 0.0 | 1.2 | 0.1 | 0.3 | 0.4 | 2.2 | 0.1 | 0.4 | 5.9 |
|  | **23B** | 0.0 | 1.6 | 0.0 | 0.2 | - | 0.4 | 0.1 | - | - |
|  | **23A/23B** | 0.1 | - | - | - | - | - | - | - | - |
|  | **24** | - | - | 0.0 | 0.8 | - | 0.9 | - | - | - |
|  | **24A** | - | 0.3 | - | - | - | - | 0.1 | - | - |
|  | **24B** | - | 0.5 | - | - | - | - | - | - | - |
|  | **24F** | - | 1.0 | 0.1 | - | - | - | 0.8 | - | - |
|  | **24/24F** | - | 2.2 | - | - | - | - | - | - | - |
|  | **25** | 0.0 | - | - | - | - | - | - | - | - |
|  | **25A** | - | 0.4 | - | - | - | - | 0.1 | - | - |
|  | **25F** | - | - | 0.0 | 0.2 | - | - | - | - | - |
|  | **25A/25F. 38** | - | - | 0.0 | 0.3 | - | - | - | - | - |
|  | **27** | - | - | - | - | - | - | 0.4 | - | - |
|  | **28A** | - | 0.2 | - | - | - | - | - | - | - |
|  | **28F** | - | - | 0.0 | 0.2 | - | - | 0.1 | - | - |
|  | **29** | - | 0.0 | 0.5 | - | - | - | 0.3 | - | - |
|  | **31** | - | 0.1 | 0.0 | - | 0.4 | - | 0.1 | - | - |
|  | **32A** | - | - | 0.0 | - | - | - | - | - | - |
|  | **33A** | - | - | - | 0.2 | - | - | 0.1 | - | - |
|  | **33B** | - | - | 0.0 | - | - | - | 0.1 | - | - |
|  | **33C** | - | - | - | - | - | - | 0.1 | - | - |
|  | **33D** | - | - | 0.1 | 0.5 | - | - | - | - | - |
|  | **34** | 0.0 | 0.1 | 0.5 | 0.3 | 0.7 | - | 0.3 | - | - |
|  | **35** | - | 0.0 | - | - | - | - | - | - | - |
|  | **35A** | - | - | 0.0 | - | - | - | 0.4 | - | - |
|  | **35B** | 0.2 | 0.8 | 0.2 | 0.8 | 0.7 | - | 0.3 | - | - |
|  | **35D** | - | - | - | 0.2 | - | - | - | - | - |
|  | **35F** | - | 0.1 | 0.1 | - | - | - | 0.8 | - | - |
|  | **35B/35D** | - | - | 0.1 | 3.4 | - | - | - | - | - |
|  | **36** | - | - | - | - | - | - | 0.1 | - | - |
|  | **38** | 0.0 | 0.6 | 0.0 | 0.3 | - | - | 0.4 | 0.4 | 0.2 |
|  | **40** | - | 0.0 | - | 0.2 | - | - | 0.1 | - | - |
|  | **41F** | - | - | - | - | - | - | 0.1 | - | - |
|  | **45** | - | - | - | - | - | - | 0.4 | - | - |
|  | **46** | - | - | 0.1 | 0.2 | - | - | - | - | - |
|  | **48** | - | - | - | - | - | - | 0.3 | - | - |
|  | ***Sub-total*** | *0.9* | *17.4* | *5.0* | *26.5* | *7.1* | *6.6* | *14.1* | *6.6* | *24.5* |
| **Undifferentiated** | **6** | 0.0 | - | - | - | - | - | - | - | - |
|  | **6A/6B/6C/6D** | - | - | - | - | - | 0.4 | - | - | - |
|  | **7** | - | - | - | - | - | 0.4 | - | - | - |
|  | **7A/7F** | - | - | 0.0 | 0.5 | - | 0.4 | - | - | - |
|  | **10A/B** | - | - | 0.0 | - | - | - | - | - | - |
|  | **11** | - | - | - | - | - | 0.4 | - | - | - |
|  | **11A/11D** | - | - | - | 0.2 | - | - | - | - | - |
|  | **11A/11D/11F** | - | - | - | 0.2 | 1.1 | - | - | - | - |
|  | **12** | - | - | 0.0 | - | - | 1.7 | - | - | - |
|  | **12A/12B/12F, 44, 46** | - | - | 0.1 | 1.4 | - | - | - | - | - |
|  | **12A/12F** | - | 0.0 | - | - | - | - | - | - | - |
|  | **12A/12F, 44, 46** | - | - | 0.0 | 1.6 | - | - | - | - | - |
|  | **15** | - | - | - | - | - | 1.7 | 0.1 | - | - |
|  | **18** | - | - | - | - | 0.7 | - | 0.1 | - | - |
|  | **18A/18B/18C/18F** | - | - | 0.0 | 1.2 | - | 0.9 | - | - | - |
|  | **19** | 0.1 | - | - | - | - | - | - | - | - |
|  | **19B/F** | - | - | 0.0 | - | - | - | - | - | - |
|  | **22** | - | - | - | - | - | 0.9 | - | - | - |
|  | **22A/22F** | - | - | - | - | 0.7 | - | - | - | - |
|  | **33** | - | 0.1 | - | 0.3 | - | - | - | - | - |
|  | **33A/33F** | - | - | - | - | - | - | 0.3 | - | - |
|  | **NVT** | 9.2 | 3.5 | 5.9 | 2.0 | - | 19.6 | 3.8 | 3.5 | - |
|  | **Pool C** | 0.0 | - | - | - | - | - | - | - | - |
|  | **Pool D** | 0.0 | - | - | - | - | - | - | - | - |
|  | **Other** | 3.4 | 7.4 | - | - | - | - | - | - | - |
|  | **Undetermined** | 2.5 | 2.7 | 0.7 | 8.3 | 7.8 | 13.9 | 3.1 | 6.2 | 4.6 |
|  | ***Sub-total*** | *15.3* | *13.8* | *6.7* | *15.6* | *10.3* | *40.4* | *7.4* | *9.7* | *4.6* |

# Supplement 4. Individual serotypes by PCV period and by WHO region – adults

| **PCV groups** | **Individual serotypes** | **AMRO** | | **AFRO** | **EMRO** | **SEARO** | | **WPRO** | |
| --- | --- | --- | --- | --- | --- | --- | --- | --- | --- |
|  |  | **Pre-PCV N=266** | **Current PCV N=1513** | **Pre-PCV N=4687** | **Current**  **PCV**  **N=60** | **Pre-PCV N=994** | **Current PCV**  **N=63** | **Pre-PCV N=157** | **Current PCV**  **N=387** |
|  |  | **%** | **%** | **%** | **%** | **%** | **%** | **%** | **%** |
| **PCV13** | ***Sub-total*** | *57.9* | *45.5* | *68.0* | *40.0* | *58.5* | *71.4* | *84.7* | *53.0* |
| **PCV20non13** | ***Sub-total*** | *11.6* | *21.8* | *9.6* | *-* | *7.6* | *11.1* | *3.1* | *10.3* |
| **nonPCV20** | **2** | - | 0.2 | - | - | 0.4 | - | - | 0.3 |
|  | **6C** | 1.1 | 2.6 | - | - | 1.0 | 3.2 | - | 0.8 |
|  | **6D** | - | 0.1 | - | - | - | - | - | - |
|  | **6F** | - | - | - | - | - | - | 0.6 | - |
|  | **7A** | - | - | - | - | 0.2 | - | - | - |
|  | **7B** | - | - | - | - | 1.0 | - | - | - |
|  | **7C** | 0.4 | 0.5 | - | - | 0.2 | - | - | - |
|  | **9A** | - | - | - | 1.7 | - | - | - | 0.5 |
|  | **9B** | - | - | - | - | 0.2 | - | - | - |
|  | **9N** | 1.5 | 3.2 | 1.9 | - | 0.6 | - | - | 0.3 |
|  | **9L/9N** | - | - | - | 3.3 | - | - | - | - |
|  | **10** | - | - | - | - | - | - | - | 0.5 |
|  | **10B** | - | - | - | - | 0.2 | - | - | - |
|  | **10C** | - | - | - | - | 0.6 | - | - | - |
|  | **10F** | - | - | - | - | 0.2 | - | - | - |
|  | **11D** | - | 0.1 | - | - | - | - | - | - |
|  | **11A/11C/11D** | - | - | - | - | - | - | - | 1.8 |
|  | **11F/11B/11C** | - | - | - | 10.0 | - | - | - | - |
|  | **12B** | - | 0.5 | - | - | 0.8 | - | - | - |
|  | **13** | 0.4 | 0.5 | 1.3 | - | 2.0 | - | - | 0.3 |
|  | **15A** | 0.8 | 2.1 | - | - | 2.0 | 1.6 | - | 9.8 |
|  | **15F** | - | - | - | - | 0.8 | - | - | - |
|  | **16** | - | 0.1 | 2.2 | - | - | - | - | - |
|  | **16F** | 0.8 | 0.6 | - | - | 0.8 | - | - | 0.3 |
|  | **17F** | 0.4 | 0.8 | - | 1.7 | 0.7 | - | - | - |
|  | **18A** | - | 0.3 | - | - | 0.4 | - | - | - |
|  | **18B** | 0.8 | - | - | - | 0.2 | - | - | - |
|  | **18F** | - | - | - | - | 0.6 | - | - | - |
|  | **19B** | - | - | - | - | 0.4 | - | - | 0.8 |
|  | **19C** | - | 0.1 | - | - | 0.4 | - | - | - |
|  | **20** | 0.8 | 0.9 | - | - | 0.4 | - | - | 1.0 |
|  | **21** | - | - | - | - | 0.4 | - | - | - |
|  | **22A** | - | - | - | - | 0.8 | - | - | - |
|  | **23A** | 0.4 | 2.1 | - | - | 1.7 | 6.3 | 3.2 | 12.1 |
|  | **23B** | 0.8 | 0.9 | - | - | 0.6 | - | - | - |
|  | **24** | - | - | - | - | 0.2 | - | - | - |
|  | **24F** | 0.4 | 0.2 | - | - | - | - | - | - |
|  | **25** | - | - | 1.4 | - | - | - | - | - |
|  | **25F** | - | 0.3 | - | - | 0.8 | - | - | - |
|  | **28A** | 0.8 | 0.4 | - | - | 0.2 | - | - | - |
|  | **28F** | - | - | - | - | 0.2 | - | - | - |
|  | **29** | - | 0.2 | - | - | 0.2 | - | - | 3.6 |
|  | **31** | - | 0.3 | - | - | 1.2 | - | - | - |
|  | **33B** | - | - | - | - | 0.2 | - | - | - |
|  | **33C** | - | - | - | - | 0.2 | - | 0.6 | - |
|  | **34** | - | 0.6 | - | - | 0.4 | 1.6 | - | 2.8 |
|  | **35A** | - | 0.3 | - | - | 0.2 | - | - | - |
|  | **35B** | - | 0.4 | - | - | 0.6 | - | - | - |
|  | **35C** | 0.4 | - | - | - | - | - | - | - |
|  | **35F** | 0.4 | 0.4 | - | - | 0.2 | - | - | - |
|  | **36** | - | - | - | - | 0.2 | - | - | - |
|  | **37** | - | 0.4 | - | - | 0.2 | - | - | - |
|  | **38** | - | - | - | - | 1.6 | - | - | - |
|  | **39** | - | - | - | - | 0.8 | - | - | - |
|  | **45** | - | - | - | - | 0.6 | - | - | - |
|  | **48** | - | - | - | - | 0.2 | - | - | - |
|  | ***Sub-total*** | *9.8* | *19.0* | *6.8* | *20.0* | *26.0* | *12.7* | *4.5* | *34.9* |
| **Undifferentiated** | **6** | - | 0.2 | - | - | - | - | - | - |
|  | **7** | - | - | - | - | - | - | 1.3 | - |
|  | **11** | - | - | - | - | - | - | - | 1.3 |
|  | **11A/11D** | - | - | - | 1.7 | - | - | - | - |
|  | **12** | - | - | - | 5.0 | - | - | - | - |
|  | **15** | - | - | - | 8.3 | - | - | - | - |
|  | **18** | - | - | - | - | - | - | 0.6 | - |
|  | **22** | - | - | - | 6.7 | - | - | - | - |
|  | **NVT** | 20.7 | 12.6 | - | 21.7 | 6.6 | - | - | - |
|  | **Other** | - | - | - | - | 0.1 | 4.8 | - | - |
|  | **Undetermined** | - | 0.9 | 15.7 | - | 1.2 | - | 5.7 | 0.5 |
|  | ***Sub-total*** | *20.7* | *13.7* | *15.7* | *43.3* | *7.9* | *4.8* | *7.6* | *1.8* |

# Supplement 5. Individual serotypes by PCV period and by WHO region – all ages

| **PCV groups** | **Individual serotypes** | **AMRO** | | **AFRO** | | **EMRO** | | **SEARO** | **WPRO** | |
| --- | --- | --- | --- | --- | --- | --- | --- | --- | --- | --- |
|  |  | **Pre-PCV N=3923** | **Current PCV N=9013** | **Pre-PCV N=4245** | **Current PCV N=5854** | **Pre-PCV N=1262** | **Current PCV N=686** | **Pre-PCV  N=302** | **Pre-PCV N=971** | **Current PCV N=617** |
|  |  | **%** | **%** | **%** | **%** | **%** | **%** | **%** | **%** | **%** |
| **PCV13** | ***Sub-total*** | *72.8* | *50.1* | *70.1* | *58.5* | *76.7* | *62.6* | *59.2* | *76.8* | *56.4* |
| **PCV20non13** | ***Sub-total*** | *10.3* | *17.6* | *6.8* | *6.2* | *7.9* | *6.6* | *2.6* | *4.7* | *13.4* |
| **nonPCV20** | **2** | - | - | 0.1 | 0.8 | 0.2 | - | - | - | - |
|  | **6C** | 1.3 | 5.5 | - | - | 0.1 | 0.3 | - | 0.2 | 0.2 |
|  | **6D** | - | 0.0 | - | - | - | 0.1 | - | - | - |
|  | **6A/6B/6C** | - | - | - | - | 0.2 | - | - | 0.3 | - |
|  | **7C** | 0.6 | 0.8 | 0.6 | 0.6 | 0.1 | 0.3 | - | - | - |
|  | **7B/7C** | - | - | - | - | - | 0.1 | - | - | 0.3 |
|  | **7B/7C, 40** | - | - | 0.0 | 0.0 | - | - | - | - | - |
|  | **9A** | - | 0.0 | - | - | 0.3 | 0.9 | - | - | - |
|  | **9B** | - | 0.0 | - | - | - | - | - | - | - |
|  | **9L** | - | 0.0 | - | - | - | 0.3 | - | - | - |
|  | **9N** | 1.6 | 2.5 | 1.0 | 0.8 | 0.6 | 0.7 | - | - | - |
|  | **9A/9L** | - | - | - | - | - | - | - | - | 0.3 |
|  | **9A/9V** | - | - | - | 0.3 | 0.5 | - | - | - | - |
|  | **9L/9N** | - | - | 0.0 | 0.2 | 0.1 | 0.6 | - | - | - |
|  | **10B** | - | - | - | - | - | 0.3 | - | - | - |
|  | **10F** | - | 0.0 | - | - | 0.1 | - | - | 0.2 | - |
|  | **10A/10F** | - | - | - | 0.0 | - | - | - | - | - |
|  | **10F/10C** | - | - | - | - | - | 0.1 | - | - | - |
|  | **10F/10C, 33C** | - | - | - | 0.1 | 0.1 | - | - | - | - |
|  | **11B** | - | - | - | - | - | - | - | - | 0.2 |
|  | **11C** | - | 0.0 | - | - | - | - | - | - | 0.2 |
|  | **11D** | - | 0.0 | - | - | - | - | - | - | - |
|  | **11A/11B** | - | - | - | 0.0 | - | - | - | - | - |
|  | **11F/11B/11C** | - | - | - | - | - | 0.4 | - | - | - |
|  | **12B** | - | 0.1 | - | - | - | 0.1 | - | - | - |
|  | **13** | 0.8 | 1.1 | 0.7 | 0.7 | 0.2 | 0.3 | - | 0.6 | - |
|  | **15A** | 0.8 | 2.0 | - | - | 0.7 | 0.7 | - | 0.6 | 12.6 |
|  | **15F** | - | 0.1 | - | - | - | 0.1 | - | - | 0.2 |
|  | **15A/15F** | - | - | - | 0.0 | - | - | - | - | - |
|  | **15A/15B/15C** | - | - | - | 0.1 | - | - | - | - | - |
|  | **16** | - | 0.0 | - | - | - | - | - | - | - |
|  | **16F** | 0.8 | 1.7 | 0.9 | 0.8 | 0.4 | 2.5 | 0.3 | - | - |
|  | **17** | - | - | - | - | 0.1 | - | - | - | - |
|  | **17A** | - | - | - | - | - | - | - | - | - |
|  | **17F** | 0.7 | 0.9 | 0.5 | 0.4 | 0.1 | 1.0 | 0.7 | 0.1 | - |
|  | **18A** | 0.7 | 0.9 | 0.0 | 0.0 | - | - | - | - | - |
|  | **18B** | 0.6 | 0.2 | - | - | - | - | - | - | - |
|  | **18F** | - | 0.0 | - | - | - | 0.3 | - | 0.1 | 0.2 |
|  | **18A/18B** | - | - | - | - | - | 0.3 | - | - | - |
|  | **19B** | - | 0.0 | - | - | - | - | - | - | 0.2 |
|  | **19C** | - | 0.0 | - | - | - | - | - | - | - |
|  | **19A/19B/19C/19F** | - | - | - | 0.2 | - | - | - | - | - |
|  | **20** | 0.8 | 1.9 | 0.0 | - | - | 0.4 | - | 0.8 | 0.0 |
|  | **21** | - | - | - | 0.1 | 0.1 | - | - | - | - |
|  | **22A** | - | - | - | - | 0.2 | - | - | - | - |
|  | **22A/22F, 38** | - | - | - | 0.0 | - | - | - | - | - |
|  | **22 non-F/A** | - | - | - | - | - | - | - | 0.6 | - |
|  | **23A** | 0.3 | 2.0 | - | - | 1.5 | 0.7 | - | 0.4 | 7.1 |
|  | **23B** | 0.9 | 1.6 | - | 0.0 | 1.1 | 0.6 | - | 0.1 | 0.3 |
|  | **24** | - | - | - | - | 0.9 | 0.1 | - | - | - |
|  | **24A** | - | 0.0 | - | - | - | - | - | - | - |
|  | **24B** | - | - | - | - | - | - | 0.3 | - | - |
|  | **24F** | 0.5 | 1.1 | - | - | 0.1 | 0.1 | - | 0.2 | - |
|  | **24A/24B/24F** | - | - | - | 0.0 | 0.2 | - | - | - | - |
|  | **24, 29** | - | - | - | 0.0 | - | - | - | - | - |
|  | **25A** | - | 0.3 | - | - | - | - | - | - | - |
|  | **25F** | - | 0.1 | 0.0 | - | - | - | - | - | - |
|  | **25A/25F, 38** | - | - | 0.2 | 0.3 | 0.2 | - | - | - | - |
|  | **26** | - | 0.0 | - | - | - | - | - | - | - |
|  | **28** | - | - | - | - | - | 0.1 | - | - | - |
|  | **28A** | - | 0.3 | - | - | 0.1 | - | - | 0.2 | - |
|  | **29** | - | 0.2 | - | - | 0.2 | 0.1 | - | - | - |
|  | **31** | - | 0.0 | - | - | 0.1 | 0.1 | - | - | - |
|  | **33A** | - | - | - | - | - | 0.1 | - | - | - |
|  | **33B** | - | - | - | - | - | - | 0.7 | - | - |
|  | **33D** | - | - | - | - | 0.1 | 0.4 | - | - | - |
|  | **33A/33F, 37** | - | - | - | 0.0 | 0.1 | - | - | - | - |
|  | **34** | 0.1 | 0.4 | 0.5 | 0.4 | 0.1 | 0.4 | - | 0.6 | 1.5 |
|  | **35A** | - | 0.1 | - | - | - | - | - | 0.8 | - |
|  | **35B** | - | 0.6 | 0.0 | 0.1 | 0.3 | 0.9 | 0.3 | 0.5 | 2.8 |
|  | **35C** | - | 0.1 | - | - | - | - | - | - | - |
|  | **35F** | 0.0 | 0.3 | 0.0 | - | 0.1 | - | - | - | - |
|  | **35A/35B** | - | - | - | 0.0 | - | - | - | - | - |
|  | **35A/35C** | - | - | - | - | - | - | - | - | 0.3 |
|  | **35A/35C, 42** | - | - | - | - | - | - | - | - | 0.2 |
|  | **37** | - | 0.0 | - | - | - | 0.1 | - | - | - |
|  | **38** | 0.1 | 0.0 | 0.1 | 0.1 | 0.2 | - | - | 0.2 | - |
|  | **39** | - | - | - | - | - | 0.1 | - | - | - |
|  | **40** | - | 0.0 | - | - | - | - | - | - | - |
|  | **42** | - | 0.0 | - | - | 0.1 | - | - | - | - |
|  | **45, 46** | - | - | - | 0.0 | - | - | - | - | - |
|  | ***Sub-total*** | *10.6* | *25.0* | *4.8* | *6.3* | *9.2* | *14.3* | *2.3* | *6.7* | *26.4* |
| **Undifferentiated** | **6** | - | 0.0 | 0.2 | - | - | - | - | - | - |
|  | **6A/6B/6C** | - | - | - | - | 0.2 | - | - | - | - |
|  | **7** | - | - | - | - | - | - | 0.7 | - | - |
|  | **7A/7F** | - | 0.0 | 0.1 | 0.4 | 0.1 | 0.1 | - | - | - |
|  | **11A/11D** | - | - | - | 0.2 | 0.6 | 0.7 | - | - | - |
|  | **12** | - | - | - | - | - | 2.2 | - | - | 0.3 |
|  | **12A/12F, 44, 46** | - | - | 0.2 | - | 0.5 | - | - | - | - |
|  | **12A/12B/12F, 44, 46** | - | - | 0.3 | 5.7 | - | - | - | - | - |
|  | **15** | - | - | - | - | - | 2.2 | - | - | - |
|  | **18** | - | 0.0 | 0.2 | - | 0.1 | 0.3 | - | - | - |
|  | **18A/18B/18C/18F** | - | - | 0.1 | 0.8 | 1.1 | - | - | - | - |
|  | **19** | - | - | - | - | - | 0.1 | - | - | - |
|  | **22** | - | - | - | - | - | 1.3 | - | - | - |
|  | **22A/22F** | - | - | - | 0.1 | 0.2 | - | - | - | 0.2 |
|  | **33A/33F** | - | - | - | - | - | 3.5 | - | - | - |
|  | **NVT** | 4.4 | 3.8 | 16.1 | 9.4 | 0.7 | 2.3 | 31.8 | 7.5 | 0.8 |
|  | **Pool G** | - | - | - | - | - | 0.1 | - | - | - |
|  | **Undetermined** | 1.9 | 3.4 | 1.1 | 12.3 | 2.6 | 3.4 | 3.3 | 4.2 | 2.4 |
|  | ***Sub-total*** | *6.3* | *7.3* | *18.3* | *28.9* | *6.1* | *16.3* | *35.8* | *11.7* | *3.7* |

# Supplement 6. Countries contributing data to figure 3.

| **PCV period** | **WHO region** | **Countries included** |
| --- | --- | --- |
| **Pediatric: 0-17 yrs** | | |
| Pre-PCV | AMRO | Costa Rica, Cuba, Guatemala, Mexico |
|  | AFRO | Algeria, Kenya, Malawi, Mozambique, South Africa, Uganda |
|  | EMRO | Iran, Tunisia |
|  | SEARO | Bangladesh, India, Nepal |
|  | WPRO | Malaysia, Philippines |
| Intermediate | AMRO | Mexico, Paraguay, Peru |
| PCV-hv | AMRO | Argentina, Brazil, Chile, Colombia, Dominican Republic, Mexico, Paraguay, Uruguay |
|  | AFRO | Burkina Faso, Cameroon, Kenya, Madagascar, Mozambique, South Africa, Togo, Zambia |
|  | EMRO | Morocco, Oman, Pakistan, Saudi Arabia |
|  | WPRO | Cambodia, Malaysia, Taiwan |
| **Adults: ≥18 yrs** | | |
| Pre-PCV | AMRO | Brazil |
|  | AFRO | South Africa |
|  | SEARO | Thailand, India |
|  | WPRO | Taiwan |
| Intermediate | WPRO | Taiwan |
| PCV-hv | AMRO | Argentina, Brazil, Colombia |
|  | EMRO | Oman |
|  | WPRO | Singapore, Taiwan |
| **All ages** | | |
| Pre-PCV | AMRO | Chile, Cuba |
|  | AFRO | Multi-country, Togo |
|  | EMRO | Iran, Lebanon |
|  | SEARO | India, Thailand |
|  | WPRO | Cambodia, Fiji, Laos |
| Intermediate | AMRO | Peru |
|  | WPRO | Taiwan |
| PCV-hv | AMRO | Brazil, Colombia, Dominican Republic, Uruguay |
|  | AFRO | Burkina Faso, Ethiopia, Ghana, Niger, South Africa |
|  | EMRO | Kuwait, Oman, Qatar, Saudi Arabia |
|  | WPRO | Taiwan |

# Supplement 7. Top 5 non-PCV20 serotypes (manuscript Table 3).

| **Age group** | **serotype** | **n** | **N** | **%** |  | **serotype** | **n** | **N** | **%** |
| --- | --- | --- | --- | --- | --- | --- | --- | --- | --- |
|  | **pre-PCV period** | | | |  | **higher valency PCV period** | | | |
| **AMRO** |  |  |  |  |  |  |  |  |  |
| Pediatric | 2 | 25 | 442 | 5.7 |  | 6C | 94 | 2747 | 3.4 |
| Pediatric | 35B | 5 | 442 | 1.1 |  | 23B | 57 | 2747 | 2.1 |
| Pediatric | 6C | 4 | 442 | 0.9 |  | 15A | 56 | 2747 | 2.0 |
| Pediatric | *15A* | *1* | *442* | *0.2* |  | 23A | 46 | 2747 | 1.7 |
| Pediatric | *15F* | *1* | *442* | *0.2* |  | 24F | 36 | 2747 | 1.3 |
| Adults | 9N | 4 | 118 | 3.4 |  | 9N | 36 | 1203 | 3.0 |
| Adults | 6C | 3 | 118 | 2.5 |  | 6C | 24 | 1203 | 2.0 |
| Adults | *15A* | *2* | *118* | *1.7* |  | 15A | 16 | 1203 | 1.3 |
| Adults | *16F* | *2* | *118* | *1.7* |  | 23A | 16 | 1203 | 1.3 |
| Adults | *18B* | *2* | *118* | *1.7* |  | 17F | 11 | 1203 | 0.9 |
| All ages | 9N | 4 | 67 | 6.0 |  | 6C | 544 | 12456 | 4.4 |
| All ages |  |  |  |  |  | 9N | 277 | 12456 | 2.2 |
| All ages |  |  |  |  |  | 15A | 209 | 12456 | 1.7 |
| All ages |  |  |  |  |  | 20 | 198 | 12456 | 1.6 |
| All ages |  |  |  |  |  | 23A | 194 | 12456 | 1.6 |
|  |  |  |  |  |  |  |  |  |  |
| **AFRO** |  |  |  |  |  |  |  |  |  |
| Pediatric | 16 | 94 | 11232 | 0.8 |  | 16F | 46 | 1924 | 2.4 |
| Pediatric | 9N | 94 | 11232 | 0.8 |  | 2 | 42 | 1924 | 2.2 |
| Pediatric | 13 | 67 | 11232 | 0.6 |  | 15A | 27 | 1924 | 1.4 |
| Pediatric | 34 | 63 | 11232 | 0.6 |  | 17F | 23 | 1924 | 1.2 |
| Pediatric | 29 | 60 | 11232 | 0.5 |  | 13 | 19 | 1924 | 1.0 |
| Adults | 16 | 103 | 4687 | 2.2 |  |  |  |  |  |
| Adults | 9N | 89 | 4687 | 1.9 |  |  |  |  |  |
| Adults | 25 | 66 | 4687 | 1.4 |  |  |  |  |  |
| Adults | 13 | 63 | 4687 | 1.3 |  |  |  |  |  |
| All ages | *38* | *2* | *266* | *0.8* |  | 9N | 126 | 8351 | 1.5 |
| All ages | *18A* | *1* | *266* | *0.4* |  | 16F | 125 | 8351 | 1.5 |
| All ages | *2* | *1* | *266* | *0.4* |  | 13 | 112 | 8351 | 1.3 |
| All ages | *20* | *1* | *266* | *0.4* |  | 7C | 85 | 8351 | 1.0 |
| All ages | *25F* | *1* | *266* | *0.4* |  | 34 | 65 | 8351 | 0.8 |
|  |  |  |  |  |  |  |  |  |  |
| **EMRO** |  |  |  |  |  |  |  |  |  |
| Pediatric | 15A | 5 | 242 | 2.1 |  | 23A | 5 | 113 | 4.4 |
| Pediatric | *34* | *2* | *242* | *0.8* |  | 16F | 3 | 113 | 2.7 |
| Pediatric | *35B* | *2* | *242* | *0.8* |  | *17F* | *2* | *113* | *1.8* |
| Pediatric | *6C* | *2* | *242* | *0.8* |  | *24* | *2* | *113* | *1.8* |
| Pediatric | *23A* | *1* | *242* | *0.4* |  | *23B* | *1* | *113* | *0.9* |
| Adults |  |  |  |  |  | *17F* | *1* | *60* | *1.7* |
| Adults |  |  |  |  |  | *9A* | *1* | *60* | *1.7* |
| All ages | 9N | 7 | 280 | 2.5 |  | 16F | 14 | 477 | 2.9 |
| All ages | 16F | 5 | 280 | 1.8 |  | 15A | 5 | 477 | 1.0 |
| All ages | 15A | 3 | 280 | 1.1 |  | 9N | 5 | 477 | 1.0 |
| All ages | 29 | 3 | 280 | 1.1 |  | 17F | 3 | 477 | 0.6 |
| All ages | 9A | 3 | 280 | 1.1 |  | 20 | 3 | 477 | 0.6 |
|  |  |  |  |  |  |  |  |  |  |
| **SEARO** |  |  |  |  |  |  |  |  |  |
| Pediatric | 10F | 8 | 623 | 1.3 |  |  |  |  |  |
| Pediatric | 24F | 6 | 623 | 1.0 |  |  |  |  |  |
| Pediatric | 35F | 6 | 623 | 1.0 |  |  |  |  |  |
| Pediatric | 2 | 5 | 623 | 0.8 |  |  |  |  |  |
| Pediatric | 7C | 5 | 623 | 0.8 |  |  |  |  |  |
| Adults | 13 | 20 | 836 | 2.4 |  |  |  |  |  |
| Adults | 15A | 20 | 836 | 2.4 |  |  |  |  |  |
| Adults | 23A | 17 | 836 | 2.0 |  |  |  |  |  |
| Adults | 38 | 16 | 836 | 1.9 |  |  |  |  |  |
| Adults | 31 | 12 | 836 | 1.4 |  |  |  |  |  |
| All ages | *17F* | *2* | *64* | *3.1* |  |  |  |  |  |
| All ages | *33B* | *2* | *64* | *3.1* |  |  |  |  |  |
| All ages | *16F* | *1* | *64* | *1.6* |  |  |  |  |  |
| All ages | *24B* | *1* | *64* | *1.6* |  |  |  |  |  |
| All ages | *35B* | *1* | *64* | *1.6* |  |  |  |  |  |
|  |  |  |  |  |  |  |  |  |  |
| **WPRO** |  |  |  |  |  |  |  |  |  |
| Pediatric | 23A | 5 | 280 | 1.8 |  | 23A | 54 | 1196 | 4.5 |
| Pediatric | 19B | 4 | 280 | 1.4 |  |  |  |  |  |
| Pediatric | 18F | 3 | 280 | 1.1 |  |  |  |  |  |
| Pediatric | 2 | 3 | 280 | 1.1 |  |  |  |  |  |
| Pediatric | *6C* | *2* | *280* | *0.7* |  |  |  |  |  |
| Adults | 23A | 5 | 157 | 3.2 |  | 23A | 39 | 300 | 13.0 |
| Adults | *33C* | *1* | *157* | *0.6* |  | 15A | 32 | 300 | 10.7 |
| Adults | *6F* | *1* | *157* | *0.6* |  | 29 | 14 | 300 | 4.7 |
| Adults |  |  |  |  |  | 34 | 12 | 300 | 4.0 |
| Adults |  |  |  |  |  | 6C | 5 | 300 | 1.7 |
| All ages | *10F* | *2* | *142* | *1.4* |  | 15A | 128 | 3690 | 3.5 |
| All ages | *24F* | *2* | *142* | *1.4* |  | 23A | 112 | 3690 | 3.0 |
| All ages | *28A* | *2* | *142* | *1.4* |  | 34 | 50 | 3690 | 1.4 |
| All ages | *34* | *2* | *142* | *1.4* |  | 35B | 50 | 3690 | 1.4 |
| All ages | *38* | *2* | *142* | *1.4* |  | 13 | 34 | 3690 | 0.9 |

n = number of cases; N = denominator; italics: serotypes with fewer than 3 cases, not shown in Table 3 in the manuscript.
